# Injectable Stimuli‐Responsive Amphiphilic Hydrogel for Rapid Hemostasis, Robust Tissue Adhesion, and Controlled Drug Delivery in Trauma and Surgical Care

**DOI:** 10.1002/adhm.202505307

**Published:** 2026-01-30

**Authors:** Arvind K. Singh Chandel, Runali Patil, Abrar Ali Khan, Deeksha Pandit, Kaushik Chatterjee, Maurice N. Collins

**Affiliations:** ^1^ School of Engineering Bernal Institute University of Limerick Limerick Ireland; ^2^ Department of Materials Engineering Indian Institute of Science Bangalore India; ^3^ Central Animal Facility Department of Materials Engineering Indian Institute of Science Bangalore India

**Keywords:** adhesive hydrogels, drug delivery, hemostatic hydrogel, injectable hydrogel, pH‐responsive hydrogel

## Abstract

Uncontrolled bleeding in trauma and surgical settings requires rapid, minimally invasive materials that can effectively stop bleeding and provide durable wound sealing. Here, we introduce an injectable, pH‐responsive amphiphilic hydrogel designed for quick hemostasis, strong wet‐tissue adhesion, and controlled therapeutic release. The hydrogel is prepared via a mild nucleophilic substitution reaction between tertiary amines of Poly(2‐(dimethylamino)ethyl methacrylate (PDMA) and gallic acidfunctionalized branched polyethyleneimine PEI(GA), using chloride‐terminated Pluronic F‐127 (Cl‐Plu‐Cl) as a crosslinker. The shear‐thinning, uniform prepolymer allows for consistent laparoscopic delivery and rapidly gels in situ (∼54 seconds) across a physiological pH range (5.07.4). In vitro and in vivo tests, including a mouse liver hemorrhage model, showed a 61% reduction in blood loss, comparable to Truseal (∼63%), while providing better injectability, biocompatibility, flexibility, and adjustable degradation and gelation properties. The (Cl‐Plu‐Cl/PDMA/PEI(GA)) hydrogel demonstrates strong adhesion strength (∼47 kPa) and withstands burst pressures up to 220 mmHg, exceeding typical arterial blood pressure. Sustained, pH‐responsive release of amoxicillin (∼60% at pH 7.4 and ∼98% at pH 5.0 over 80 hours) displayed antibacterial activity against Staphylococcus aureus and MRSA. Alamar Blue and Live/Dead assays confirmed over 90% cell viability, and the gradual in vitro degradation over three weeks indicates safe resorption and potential for clinical use.

## Graphical abstract

**Fast‐acting hydrogel seals bleeding wounds** as the illustrated injectable, pH‐responsive network rapidly gels in situ to stop hemorrhage, adhere strongly to wet tissue, and release antibiotics in a controlled, pH‐dependent manner. The material withstands high pressures, shows excellent biocompatibility, and degrades safely, offering a versatile platform for surgical wound management.

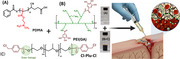

## 1 Introduction

Uncontrolled hemorrhage remains a leading cause of morbidity and mortality in trauma, surgery, and battlefield injuries. This highlights the urgent need for advanced, innovative hemostatic biomaterials that offer rapid, effective, and adaptable bleeding control, particularly in high‐flow, complex injury environments [1, 2, 3]. Conventional hemostatic agents, including traditional dressings, sponges, sutures, and fibrin‐based sealants, often underperform in dynamic physiological settings owing to their limited adhesiveness and injectability, slow gelation kinetics, and reliance on external initiators such as UV light, catalysts, or photo‐responsive systems [4, 5, 6, 7, 8, 9, 10, 11, 12]. Additionally, these materials often fail to perform effectively in dynamic physiological environments, where irregular wound geometries, continuous tissue motion, and high‐volume bleeding, as well as gravity‐induced displacement, challenge their functional stability and retention on site [13]. Furthermore, their poor tissue adhesion, insufficient mechanical strength, and limited tunability restrict their applicability and efficacy in severe hemorrhage scenarios, highlighting the urgent demand for next‐generation hemostatic materials that combine rapid action, robust adhesion, and minimally invasive delivery [7, 14]. A brief overview of representative commercial hemostatic sealants and dressings, including their compositions, advantages, limitations, and reported mechanical performance (adhesion and burst pressure), is provided in Table S1, further illustrating these shortcomings. Collectively, these limitations emphasize the need for injectable hemostatic systems that can rapidly form a stable in‐situ gel, adhere strongly to wet and dynamically moving tissues, and maintain sufficient mechanical integrity to withstand physiological pressures while ensuring durable wound sealing over clinically relevant timescales.

pH‐responsive cationic biomaterials‐based hydrogels have gained attention for their ability to electrostatically interact with negatively charged blood and tissue components, accelerating coagulation and enhancing adhesion [15, 16, 17]. Among these, poly(2‐dimethylaminoethyl methacrylate) (PDMA) is one of the most extensively studied dual‐responsive polymers, exhibiting pH‐ and temperature‐sensitivity. At injury sites, where the local microenvironment is typically slightly acidic, protonation of PDMA enhances its interactions with anionic phospholipids and facilitates erythrocyte aggregation and platelet activation, ultimately supporting rapid fibrin clot formation [16, 18]. Despite these advantages, PDMA‐based hydrogels often display insufficient intrinsic bioadhesion, making them prone to displacement under high‐flow or mechanically dynamic conditions, which in turn compromises their hemostatic efficiency [19]. To overcome these challenges, next‐generation hydrogel systems must integrate stronger tissue adhesion and improved mechanical stability to ensure robust performance in complex physiological environments. However, existing PDMA‐based hydrogels and related adhesive systems mainly focus on enhancing bulk mechanics or general hydrogel‐hydrogel adhesion and do not simultaneously offer strong wet tissue adhesion, high burst resistance, and pH‐tunable therapeutic release within an injectable platform explicitly designed for rapid hemostasis under actively bleeding conditions.

pH‐responsive poly(2‐dimethylaminoethyl methacrylate) (PDMA)‐based hydrogels have gained interest because of their cationic properties, which allow electrostatic interactions with negatively charged blood and tissue components, thereby aiding coagulation at slightly acidic injury sites. However, traditional PDMA networks often lack sufficient natural bioadhesion and possess limited mechanical strength, making them susceptible to displacement and failure under high‐flow or dynamic mechanical conditions. Past approaches to enhance PDMA‐based hydrogels, such as adding ionic comonomers, grafting PDMA onto cellulose backbones, or adding nanoparticle reinforcements, mainly improve bulk mechanical properties or general hydrogel‐hydrogel adhesion, but do not concurrently achieve strong wet tissue adhesion, high burst resistance, and pH‐controlled antibiotic release within an injectable platform tailored for rapid hemostasis [20, 21, 22, 23, 24].

To address these limitations, we present a novel injectable hydrogel system derived from PDMA and gallic acid (GA)‐conjugated branched polyethyleneimine (PEI). It is crosslinked via a strategically designed, activated chloride‐terminated Pluronic F‐127 (Cl‐Plu‐Cl) linker. Our hydrogel exhibits thermoresponsive behavior, and the incorporation of ester bonds ensures controlled biodegradation. PEI, widely studied as a cationic vector for gene delivery, and GA, recognized for its strong adhesive and antioxidant properties, act synergistically to enhance both hemostatic and functional performance [25, 26]. In particular, GA exhibits potent antioxidant activity, enabling the scavenging of reactive oxygen species (ROS) typically generated during injury and inflammation, thereby improving biocompatibility and potentially accelerating wound healing by reducing oxidative stress. Recent studies indicate that while transient ROS bursts assist host defence and signalling, sustained ROS overproduction leads to oxidative damage, chronic inflammation, and delayed or non‐healing wounds; therefore, ROS‐scavenging biomaterials can restore redox balance, protect cells, and facilitate proper progression through the wound‐healing phases [27, 28, 29]. Motivated by these features, the hydrogel was purposely designed to combine rapid haemostasis and strong tissue adhesion with inherent ROS‐scavenging ability. A DPPH assay confirmed that the PEI(GA) component offers measurable antioxidant activity. Building on these features, our multi‐component Cl‐Plu‐Cl/PDMA/PEI(GA) hydrogel architecture is designed to deliver synergistic hemostatic and bioadhesive properties while providing tunable gelation kinetics, predictable degradation, and the potential for sustained drug release. Notably, the hydrogel's injectability allows for effective delivery to irregular or deep wound sites, ensuring close contact with bleeding tissues, which is an advantage over conventional dressings and sealants. The combined cationic charge and GA‐mediated adhesion further improve tissue retention and resistance to displacement under blood flow, creating a robust, multifunctional platform for advanced hemorrhage control.

The high cationic charge density contributed by both PDMA and dendritic PEI facilitates strong electrostatic interactions with negatively charged blood components, including RBCs, and platelet activation, thereby accelerating fibrin clot formation [30]. Simultaneously, GA‐functionalized PEI provides robust tissue adhesion through a combination of hydrogen bonding, electrostatic interactions, and hydrophobic effects, effectively mimicking the wet‐surface adhesion mechanisms of marine mussel proteins [31]. This dual functionality ensures firm hydrogel anchorage to tissue surfaces, even under highly moist physiological conditions, a critical requirement for efficient hemostasis. Notably, the gelation process proceeds via a nucleophilic substitution reaction, which occurs under mild aqueous conditions without the need for external initiators or generation of cytotoxic byproducts, thereby enhancing biocompatibility and simplifying in situ application [16, 19]. Furthermore, the hydrogel exhibits a set of advanced, tunable properties including adjustable gelation kinetics and mechanical strength, biodegradability through ester linkages within the PEG‐based crosslinker, and dual hemostatic mechanisms mediated by both cationic charge‐induced coagulation and GA‐enhanced tissue adhesion [32, 33]. Such structural programmability enables controlled clearance in vivo, minimizing the risk of long‐term accumulation. Overall, the combination of injectability, robust adhesion, mechanical adaptability, and safe, initiator‐free gelation represents a substantial improvement over conventional hemostatic agents, offering a multifunctional platform for effective hemorrhage control in complex wound environments.

**FIGURE 1:**
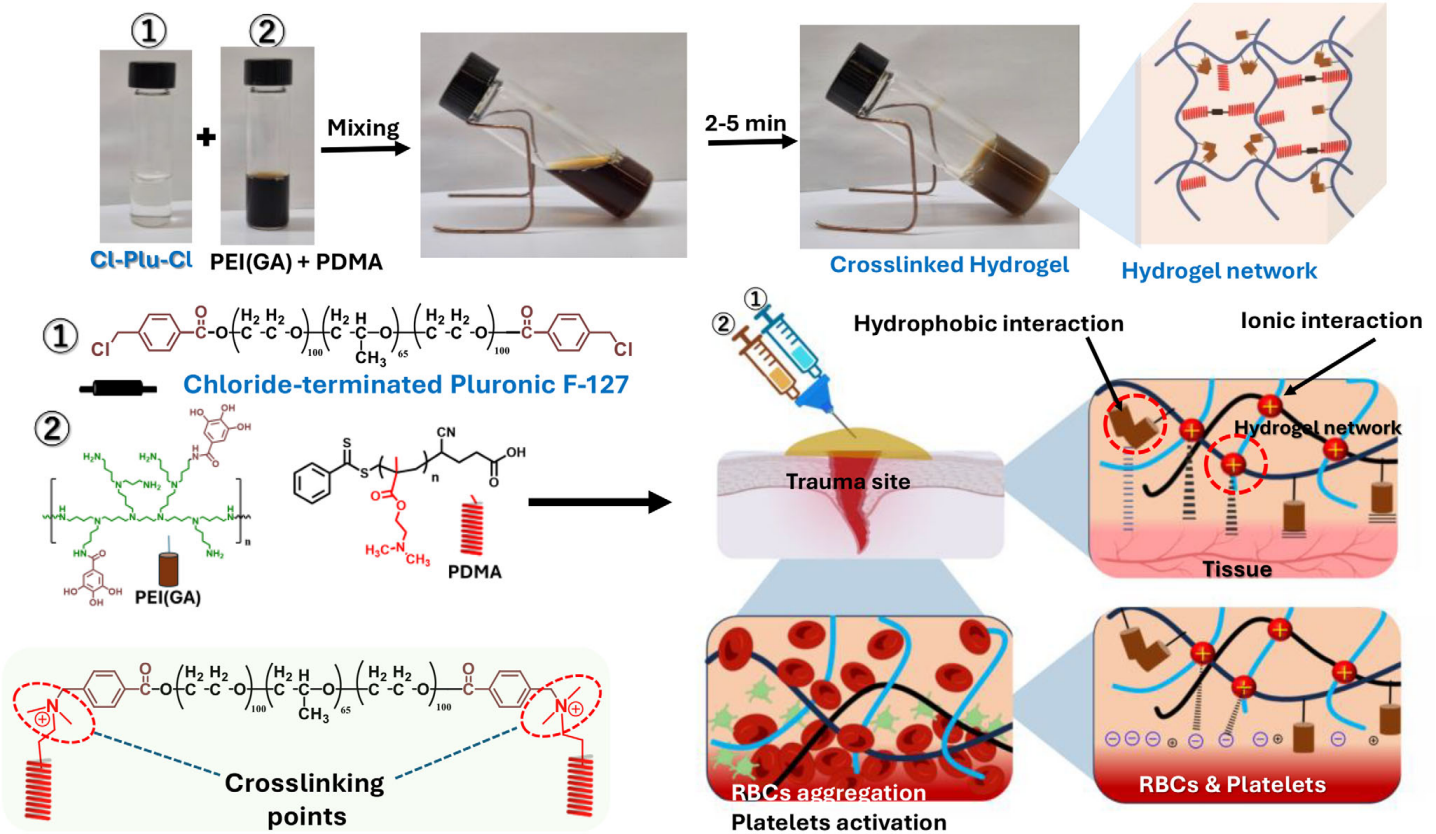
Schematic illustration of the crosslinked, injectable, pH‐responsive PDMA/PEI(GA)/Cl‐Plu‐Cl hydrogel designed as a hemostatic and tissue adhesive.

We hypothesized that incorporating gallic acid–functionalized dendritic polyethyleneimine (PEI(GA)) into a pH‐ and temperature‐responsive PDMA network, crosslinked with degradable chloride‐terminated Pluronic F‐127 (Cl‐Plu‐Cl), would give the injectable hydrogel strong wet tissue bioadhesion while maintaining injectability, cytocompatibility, and hemostatic performance. The high cationic charge density of PDMA and PEI is expected to improve blood coagulation (Figure 1). Meanwhile, GA offers mussel‐inspired adhesion and antioxidant activity, and the Pluronic‐based crosslinker provides mechanical stability and controlled biodegradation. Accordingly, this study aimed to develop and thoroughly evaluate an injectable, pH‐responsive Cl‐Plu‐Cl/PDMA/PEI(GA) hydrogel for rapid hemostasis, durable wet tissue adhesion, and controlled antibiotic release, and evaluation of antimicrobial test with *S. aureus* and MRSA strains. To accomplish this, we systematically examined the hydrogel's gelation behavior, rheological and mechanical properties, swelling and degradation, adhesion and burst pressure, cytocompatibility and hemocompatibility, antioxidant activity, pH‐dependent amoxicillin release, in vitro coagulation performance, and in vivo hemostatic efficacy in a mouse liver bleeding model.

## 2 Materials and Methods

### 2.1 Materials

Pluronic F‐127 (Mn 12 600 g/mol), 4‐chloromethylbenzoyl chloride (Cl‐Bz‐Cl, 98%), polyethylenimine, branched (PEI, Mn 60 000 g/mol), the RAFT agent S‐1‐dodecyl‐S′‐(α,α′‐dimethyl‐α″‐acetic acid) trithiocarbonate, 2‐dimethylaminoethyl methacrylate (DMAEMA), gallic acid (GA), and phosphate‐buffered saline (PBS, 98%) were purchased from Sigma‐Aldrich (St. Louis, MO, USA). 4‐(4,6‐Dimethoxy‐1,3,5‐triazin‐2‐yl)‐4‐methylmorpholinium chloride (DMTMM) was obtained from Fluorochem. Dialysis membranes (Spectra/Por 1, MWCO 6–8 kDa) were purchased from Spectrum Laboratories, Inc. (Rancho Dominguez, CA, USA). Deuterium oxide (D_2_O), deuterium chloride solution (DCl), low‐glucose Dulbecco's Modified Eagle Medium (DMEM), penicillin‐streptomycin‐amphotericin‐B suspension (PSA), fetal bovine serum (FBS), penicillin‐streptomycin solution, and trypsin‐EDTA were obtained from Thermo Fisher Scientific. 1,1‐Diphenyl‐2‐picrylhydrazyl (DPPH), Triton X‐100, and all other reagents were of analytical grade and used as received without further purification. The L‐929 mouse fibroblast cell line was obtained from the American Type Culture Collection (ATCC, USA) and cultured according to the supplier's instructions.

### 2.2 Methods

#### 2.2.1 Synthesis of PDMA

The synthesis of PDMA was performed according to our previously reported procedure [16]. In brief, 2‐(Dimethylamino)ethyl methacrylate (DMA, 20 g) was dissolved in N, N‐dimethylformamide (DMF, 20 mL) at ambient temperature. The RAFT agent S‐1‐dodecyl‐S′‐(α,α′‐dimethyl‐α″‐acetic acid) trithiocarbonate (0.6 g, 1.64 × 10^−^^4^ mol) was added, and the solution was degassed by purging with nitrogen for 20 min. Azobisisobutyronitrile (AIBN, 0.016 g, 9.6 × 10^−^^5^ mol) was then introduced under an argon atmosphere, and the reaction vessel was sealed with a rubber septum. Polymerization was carried out at 70 °C with continuous stirring for 10 h, achieving a monomer conversion of approximately 83%. Upon completion, the reaction mixture was cooled to room temperature and precipitated in excess hexane. The solvent was removed by rotary evaporation, and the crude polymer was washed with hexane and dried under vacuum at 60°C for 24 h. Gel permeation chromatography (GPC) analysis revealed an average molecular weight of 27 000 g/mol with a polydispersity index (PDI) of 1.3. This macro‐RAFT initiator is hereafter referred to as PDMA‐RAFT.

#### 2.2.2 Synthesis of PEI(GA)

The conjugation of branched polyethyleneimine (PEI) with gallic acid (GA) was performed through a carbodiimide‐ mediated reaction. Briefly, polyethyleneimine (2 g, M_n_ ∼60 kDa) was dissolved in 100 mL of dimethyl sulfoxide (DMSO) in a 250 mL flask. Separately, gallic acid (1. 35 g) was dissolved in 10 mL of DMSO in a 50 mL round‐ bottom flask. 4‐(4, 6‐ Dimethoxy‐ 1, 3, 5‐ triazin‐ 2‐ yl)‐ 4‐methylmorpholinium chloride (DMTMM), 2.20 g was added to the gallic acid solution, and the mixture was stirred at room temperature for one hour. The activated gallic acid solution was then added dropwise to the PEI solution, and the mixture was stirred for 16 h at room temperature. Afterward, distilled water (40 mL) was added, and the crude product was dialyzed against a 0.1 m NaCl solution, followed by distilled water using an 8–14 kDa molecular weight cut‐off (MWCO) dialysis membrane. Following the initial salt wash, dialysis continued in distilled water with periodic changes over four days. The resulting solution was freeze‐dried to produce a foamy PEI(GA) conjugate, stored in an airtight container at 4°C until further use. The chemical structure of PEI(GA) was confirmed via Fourier‐ transform infrared spectroscopy (FT‐IR), proton nuclear magnetic resonance (^1^H NMR) spectroscopy, and UV–vis spectroscopy. PEI(GA) was synthesized by coupling gallic acid to branched PEI (60 kDa) using DMTMM in DMSO, followed by dialysis and lyophilization as described above. Quantitative ^1^H NMR analysis in D_2_O was employed to determine the degree of substitution (DS) by comparing the integral of gallic acid's aromatic protons at 6.6.8–7.2 ppm with that of PEI's methylene protons at 2.5–3.0 ppm, assuming one amine unit as –CH_2_–CH_2_–NH–(4 H). The resulting DS was approximately 21% of primary amines. Independent UV–vis analysis, using a gallic acid calibration curve (λ_max_ ≈ 260–270 nm) on aqueous PEI(GA) solutions, yielded a closely matching DS of 19.4%, confirming the grafting level, Figure S1.

#### 2.2.3 Synthesis of Cl‐Pluronic F‐127‐Cl (Cl‐Plu‐Cl) Crosslinker

The halide‐terminated Pluronic F‐127 crosslinker (Cl‐Plu‐Cl) was synthesized via esterification, as previously described [16, 19]. In this procedure, Pluronic F‐127 (15 g, M_n_ ∼12,600 g/mol; Aldrich) was dissolved in dry toluene (250 mL) under a nitrogen atmosphere. 4‐(Chloromethyl)benzoyl chloride (Cl‐BzCl, 1.875 g, 9.75 × 10^−^^3^ mol) was added to the solution, and the flask was sealed with a rubber septum. The reaction mixture was cooled in an ice bath. Triethylamine (1.35 mL, 9.75 × 10^−^^3^ mol) was then added dropwise via syringe under nitrogen protection. The reaction was allowed to proceed at ambient temperature for 12 h with continuous stirring. Upon completion, the reaction mixture was filtered to remove the precipitated byproduct. The solvent was evaporated under reduced pressure at 60°C using a rotary evaporator. The crude product was redissolved in deionized water and centrifuged at 5000 rpm for 10 min to remove any residual insoluble material. The clear supernatant was collected and lyophilized to obtain the final Cl‐Plu‐Cl crosslinker. The chemical structure of Cl‐Plu‐Cl was confirmed by proton nuclear magnetic resonance (^1^H, NMR) spectroscopy. Chloromethyl‐terminated pluronic F‐127 (Cl‐Plu‐Cl) was prepared by esterification of F‐127 with 4‐chloromethylbenzoyl chloride in dry toluene using triethylamine as a base, followed by purification and lyophilization. The degree of end‐group functionalization was measured by ^1^H NMR in D_2_O, based on the ratio of benzoyl aromatic protons (7.4–8.1 ppm) to PPO/PEO methylene protons (3.2–3.8 ppm), indicating approximately 99%–100% (Figure S1d), 4‐chloromethylbenzoyl substitution per Pluronic chain. This high degree of functionalization confirms the effectiveness of the crosslinker synthesis.

#### 2.2.4 Preparation of an Injectable Hydrogel System

Lyophilized Cl‐Plu‐Cl powder was dissolved in phosphate‐buffered saline (PBS, pH 7.4) at a concentration suitable to achieve the desired final polymer content. Similarly, PDMA was dissolved in PBS (pH 7.4) at the required concentration for hydrogels containing dendritic polyethyleneimine‐gallic acid (PEI (GA)). PEI(GA) was dissolved separately in PBS to obtain a stock solution. The Cl‐Plu‐Cl, PDMA, and PEI(GA) solutions were then combined in specific weight ratios (w/w/w) as listed in Table 1. The final hydrogel formulations were adjusted to maintain a total polymer concentration of 20% (w/v) for the cross‐linked Cl‐Plu‐Cl/PDMA network, with PEI(GA) added at concentrations ranging from 0.5% to 3% (w/v). Each mixture was vortexed vigorously for 10–15 s and incubated at either 25°C or 37°C. Gelation was monitored visually by vial inversion, and gelation kinetics were further characterized by using a rheometer. The resulting prepolymer solutions were readily injectable through a hypodermic syringe. Four formulations, Gel‐1, Gel‐2, Gel‐3, and Gel‐4, with defined Cl‐Plu‐Cl:PDMA: PEI(GA) ratios (w/w/w), were prepared, ensuring that the total polymer content and PEI(GA) loading remained constant across all samples.

**TABLE 1:** Composition, observed gelation time, sol fraction, and equilibrium water swelling (*S*_w_) of the hydrogels.

Hydrogel	Cl‐Plu‐Cl/PDMA PEI(GA)(w/w/w)	Gelation time(s)at 25°C/37°C	Equilibrium Swelling(%) at 37°C		Sol‐fraction (%, w/w)	
			pH 7.4	pH 5.0	PBS	DMF
Gel‐1	4/6/0.01	102/54	333(±75)	420(±16)	2.1(±0.6)	2.75(±0.2)
Gel‐2	3/7/0.01	158/82	554(±12)	773(±105)	4.2(±0.45)	5.2(±0.3)
Gel‐3	2/8/0.01	268/149	791(±38)	969(±81)	4.8±0.15)	5.6(±0.36)
Gel‐4	4/6/0.00	119/66	296(±37)	412(±49)	1.8(±0.3)	2.2(±0.2)

#### 2.2.5 Characterization of the Hydrogels

The hydrogels were characterized by FT‐IR spectroscopy (PerkinElmer, United States). Sol fraction, water swelling, and degradation experiments were performed using standard protocols. The gelation time of the Cl‐Plu‐Cl/PDMA/PEI(GA) hydrogels with varying weight ratios was measured using a protocol adapted from a previously reported method [34]. Briefly, 20% w/v,100 µL of the PDMA/PEI(GA) solution was placed in a 30 mm diameter plastic dish (Thermo Scientific Sterilin) under continuous stirring at 300 rpm. Subsequently, 100 µL of the 20% w/v Cl‐Plu‐Cl solution, with or without the addition of PEI(GA), was added to the dish. The gelation time was defined as the interval between the addition of the crosslinker solution and the formation of a solid, non‐flowing globule. For each hydrogel formulation, gelation times were determined in quadruplicate (*n*  =  4) and reported as mean ± standard deviation. The morphology of the hydrogels was analyzed by lyophilization and SEM (Hitachi SU70 microscope, Hitachi High‐Tech Corporation, Japan) at 10 kV and JSM‐IT300 JEOL Ltd at 15 kV.

#### 2.2.6 Rheological Properties and Evaluation of Gelation Time

Rheological measurements were performed using a Discovery Hybrid Rheometer (DHR‐2, TA Instruments, Delaware, USA) equipped with a 25 mm parallel plate geometry. Before testing, the instrument was calibrated for inertia, friction coefficient, and plate alignment. Unless otherwise stated, all measurements were conducted at 25°C, and a liquid trap was used to prevent water evaporation during testing. Viscosity was assessed as a function of shear rate over a range of 1 to 1000 s^−^^1^ to evaluate shear‐thinning behavior. For further analysis of mechanical properties, strain sweep tests were performed at a fixed angular frequency of 5.0 rad s^−^^1^ across a strain range of 0.0125 % to 100 % to determine the linear viscoelastic region (LVR). Storage modulus (G') and loss modulus (G″) were recorded throughout. The gap between the plates was set to 0.1 mm for time‐ and frequency‐sweep tests and to 0.2 mm for temperature‐sweep experiments to accommodate fully swollen hydrogel samples.

For gelation kinetics, time sweep experiments were conducted at 25°C and 37°C, using a constant strain of 1% and a frequency of 1 Hz, within the LVR. Prepolymer solutions (Cl‐Plu‐Cl and PDMA/PEI(GA)/ (also in the presence of whole sheep blood), each at 20% w/v in PBS, final polymer content 20%) were mixed. They were immediately injected (300 µL) between the parallel plates. A 15–20 s time delay occurred between mixing and loading. Gelation time was defined as the crossover point where G' matched G“ at the set temperature. Frequency sweep measurements were conducted at 37°C, 30 min post‐gelation, over an angular frequency range of 0.1 to 100 rad s^−^^1^ at a fixed strain of 1%. For temperature‐dependent behaviour, temperature sweeps were performed at 25°C and 37°C using freshly prepared hydrogel samples. To further analyze mechanical robustness, G' and G” were also measured across a strain range of 10^−^^2^ to 10^3^ % at ω = 6.3 rad/s, and across a frequency range of 10^−^^1^ to 10^2^ rad/s at *γ* = 5 %. Self‐healing properties were evaluated by alternating between low (1%) and high (200%) shear strains at an angular frequency of 6.3 rad/s, each held for 200 s. Shear‐thinning behavior was also assessed over a wide shear rate range of 10^−^^2^ to 10^3^ s^−^^1^.

#### 2.2.7 Adhesion Test

The adhesive properties of the prepared hydrogels were evaluated qualitatively on various substrates, including human skin, chicken skin, bone, metal, rubber, and glass, as shown in the results and discussion section. Approximately 300 µL of the Cl‐Plu‐Cl/PDMA/PEI(GA) prepolymer solution (20 wt% total polymer) was applied between two substrates and allowed to undergo in situ gelation at 37°C for 5–10 min under gentle contact to form a cohesive hydrogel layer. After gelation, the adhered assemblies were lifted, tilted, and gently shaken to visually assess whether the hydrogel could maintain contact and support the weight of the attached substrate without detachment, representative images were captured immediately after lifting and during manipulation to illustrate the qualitative adhesion behavior. For experiments involving human finger visuals to demonstrate adhesion, written informed consent was obtained from the participant before the study, in accordance with ethical guidelines. The assessment was purely qualitative and not force‐quantified. It was intended to demonstrate the hydrogel's ability to adhere to diverse biological and non‐biological surfaces relevant to surgical and wound environments.

#### 2.2.8 Peeling Adhesion Test

The peeling adhesion test was performed according to the reported methods [35]. The sausage skin membrane was fixed in place with a clamp. Then, Cl‐Plu‐Cl and PDMA/PEI(GA) hydrogel solutions (1 mL) were injected onto the membrane surface (80 × 20 mm). The hydrogel was allowed to complete crosslinking for one hour at room temperature (to avoid dehydration, kept in a moisture chamber), as illustrated in the results and discussion part. Using an IMADA ZTA‐005 N load cell (IMADA Co., Ltd, Japan), the peel adhesion test was performed in unidirectional tension, while recording the force and the extension. The loading rate was kept constant at 1 mm/min. All measurements were repeated three times.

#### 2.2.9 In Vitro Lap Shear Test

The lap shear strength of the Cl‐Plu‐Cl and PDMA/PEI(GA) hydrogels was evaluated following a modified ASTM F2255‐05 standard, as adapted from a previously reported method [35, 36]. Sausage casing membranes were cut into rectangular strips (40 × 10 mm^2^) and gently washed three times with phosphate‐buffered saline (PBS, pH 7.4). A 50 µL volume of the hydrogel solution was applied to the central 10 × 10 mm^2^ area of one membrane, and a second membrane was placed over it to form an overlapping adhesion interface. The assembled samples were incubated at 37°C for 1 h in a humidified chamber to allow complete crosslinking while preventing dehydration. After curing, the samples were mounted in an IMADA ZTA‐005N universal testing machine (IMADA Co. Ltd, Japan). Shear testing was performed in tension mode at a constant strain rate of 1 mm/min until failure occurred. The peak force recorded at the point of detachment was used to determine the lap shear strength. Each test was repeated in quadruplicate (*n* = 4) for statistical analysis. To assess the impact of pH on interfacial adhesion, lap‐shear tests were also performed after pre‐equilibrating hydrogels and tissue substrates in buffer solutions at pH 5.0 or pH 7.4.

#### 2.2.10 Burst Pressure Test

Burst strength measurements of the Cl‐Plu‐Cl and PDMA/PEI(GA) hydrogels were conducted following the standard protocol of the American Society for Testing and Materials (ASTM F2392‐04) [32]. In this study, porcine sausage skin served as an ex vivo tissue model to simulate the mechanical properties of biological membranes. The porcine sausage skin casing was cut into circular samples with a 30 mm diameter and prepared with a central 2 mm pinhole, as illustrated. The pre‐gel solutions were prepared as previously described and poured onto the porcine skin samples, which were supported by a silicone disc (2 mm thick, 15 mm inner diameter) to ensure even placement. The hydrogels were cross‐linked in situ by incubating the samples at 37°C for 30 min under sealed conditions. The prepared porcine skin specimens with the cross‐linked hydrogel sealant were then mounted onto the burst strength measurement apparatus. Saline was introduced beneath the tissue model at a steady flow rate of 2 mL min^−^^1^. The maximum internal pressure at which the hydrogel ruptured, either laterally or at the pinhole site, was recorded as the burst strength. All measurements were performed in triplicate for each formulation, and results were expressed as mean ± standard deviation.

#### 2.2.11 Evaluating the Cytotoxicity of Cl‐Plu‐Cl/PDMA/(PEI(GA) Hydrogels

The cytocompatibility of the synthesized Cl‐Plu‐Cl/PDMA/PEI(GA) hydrogels was assessed using the Alamar Blue assay. L929 fibroblast cells (ATCC, USA) were cultured in Dulbecco's Modified Eagle Medium (DMEM; Sigma–Aldrich, Ireland) supplemented with 10% fetal bovine serum (FBS), 1% penicillin‐streptomycin, and 1% L‐glutamine. Cells were maintained under standard conditions (37°C, 5% CO_2_, humidified atmosphere), and the media were refreshed twice a week until the cells reached 80% confluence. Subculturing was performed before experimental seeding. For the experiments, L929 cells were seeded into 24‐well plates at a density of 5 × 10^4^ cells per well and allowed to adhere for 24 h. For hydrogel‐based testing, preformed Cl‐Plu‐Cl/PDMA/PEI(GA) hydrogels were sterilized by UV exposure for two hours before use, followed by equilibration in complete culture medium. Hydrogels (50–400 mg) were then transferred into wells either in direct contact with the cells or placed into Transwell inserts (Corning Inc., USA) to evaluate indirect contact cytotoxicity, as illustrated in Figure S2.

For extract (solution‐phase) cytotoxicity assays, fully crosslinked Cl‐Plu‐Cl/PDMA/PEI(GA) hydrogels were immersed in complete DMEM at a hydrogel loading of 5% (w/v) and incubated at 37°C for 24 h to allow leaching of non‐crosslinked species (sol‐fraction) into the medium. The resulting hydrogel extracts were collected and diluted with fresh complete DMEM to obtain solutions corresponding to nominal extract concentrations of 0.1%, 0.2%, 0.5%, and 1.0% (w/v), which were then added to L929 cell‐seeded wells and incubated for 48 h under standard culture conditions. After incubation, the medium was replaced with fresh medium containing 10% (v/v) Alamar Blue, and cells were further incubated for two hours at 37°C. Absorbance was measured at 570 nm with 600 nm as the reference wavelength using a microplate reader (SpectraMax iD3, United States), and cell metabolic activity was quantified from the reduction of resazurin to resorufin, with higher 570 nm signals indicating greater cell viability; cell viability (%) was calculated by normalizing the absorbance of each test group to the mean absorbance of untreated control wells using Equation (1):

(1) $$\mathrm{Cell}\;\mathrm{Viability}\;(\%)=\frac{\mathrm{Ab}s_{\mathrm{sample}}-\mathrm{Ab}s_{\mathrm{blank}}}{\mathrm{Ab}s_{\mathrm{control}}-\mathrm{Ab}s_{\mathrm{blank}}}\;X100$$

where Abs_sample_, Abs_blank_, and Abs_control_ indicate the absorbance values of the sample well, blank well (containing only culture medium), and control well (cells without test material), respectively. For qualitative visualization of cell viability, a live/dead staining assay was also performed by incubating NIH 3T3 cells with calcein‐AM (2 µm) and ethidium homodimer‐III (4 µm) for 30 min at 37°C, followed by imaging with a confocal laser scanning microscope (IX‐53, Olympus, Japan). Additional procedures for collecting hydrogel degradation products under physiological conditions, characterizing them by FT‐IR and dynamic light scattering, and evaluating their cytocompatibility and hemocompatibility using Alamar Blue and hemolysis assays are described above. All experiments were performed in quadruplicate (*N*  =  4) for each polymer solution and hydrogel formulation, and results are reported as mean ± standard deviation.

#### 2.2.12 Radical Scavenging Activity

The free radical scavenging activity of the synthesized Cl‐Plu‐Cl/PDMA/PEI(GA) hydrogels was evaluated using a 2,2‐diphenyl‐1‐picrylhydrazyl (DPPH) assay adapted from a previously reported protocol [37]. Briefly, DPPH was dissolved in methanol to prepare a stock solution (50 µm). For solution‐phase testing, 50 µL of DPPH solution was mixed with 50 µL of a series of PEI(GA) polymer solutions at concentrations of 0, 1.6, 3.1, 6.3, 12.5, 25, 50, and 100 mg mL^−^^1^. Ascorbic acid (AA, 50 mm) was used as a positive control. The mixtures were incubated in the dark on a shaker at 200 rpm for 30 min. The absorbance was measured at 517 nm using a SynergyMx (BioTek, UK). The DPPH solution (25 µm) served as the 100% radical control. The residual DPPH concentration was determined from a standard calibration curve, and the radical scavenging activity (%) was calculated as previously described [38].To assess the radical scavenging activity of the crosslinked hydrogels, 100 µL hydrogel discs (Cl‐Plu‐Cl/PDMA with or without PEI(GA); final polymer concentration 20% w/v with 0.01% w/v PEI(GA) were immersed in 150 µL DPPH solutions at different concentrations (25, 50, and 100 µm). Additionally, the effect of varying crosslinker or PEI(GA) content was evaluated by preparing hydrogels with different PEI(GA) loadings or polymer ratios. The hydrogel‐DPPH mixtures were incubated in the dark on a shaker at 200 rpm for 30 min. After incubation, the absorbance of the DPPH solution was measured at 517 nm, and the radical scavenging activity (%) was calculated accordingly. All measurements were performed in triplicate, and results were reported as mean ± standard deviation.

#### 2.2.13 Mechanical Properties

The compressive mechanical properties of the Cl‐Plu‐Cl/PDMA/PEI(GA) hydrogels were evaluated using uniaxial compression testing. Uniform, cylindrical, fully cross‐linked hydrogel samples were prepared by casting prepolymer solutions into custom‐made molds with a 10 mm diameter and a 10 mm depth. After overnight cross‐linking at 37°C, the hydrogels were incubated in phosphate‐buffered saline (PBS, pH 7.4) at 37°C for 24 h to achieve equilibrium swelling. For pH‐dependent measurements, fully swollen hydrogels were additionally equilibrated for 24 h in either PBS (pH 7.4) or acetate buffer (pH 5.0) at 37°C before testing. Uniaxial compression tests were performed using a tensile–compressive universal testing machine (IMADA Co., Ltd., Japan) equipped with an IMADA ZTA‐005N load cell. Each hydrogel specimen was positioned between two parallel compression plates, and the upper plate was lowered at a constant crosshead speed of 1 mm min^−^^1^ until 75 % strain relative to the initial sample height. The height and diameter of each hydrogel sample were measured prior to testing using a Mitutoyo Absolute Digimatic thickness gauge to ensure accurate dimensional data. During testing, force (N) and displacement (mm) data were continuously recorded and converted into engineering stress–strain curves for each sample. The compressive modulus E_c_ (kPa) under uniaxial loading was determined from the slope of the linear region of the stress–strain curve between 15%–25% strain. Assuming nearly incompressible behavior typical of highly swollen hydrogels (ν ≈ 0.5), the corresponding Young's modulus E was estimated from the compressive/shear modulus using E  =  3E_c_. The maximum compressive stress at failure was also determined for each sample. Three replicate measurements were performed for each hydrogel formulation, and all values are reported as mean ± standard deviation (*n*  =  3).

#### 2.2.14 Swelling and Degradation of the Hydrogels

The swelling behavior of the synthesized Cl‐Plu‐Cl/PDMA/PEI(GA) hydrogels was evaluated gravimetrically. Briefly, the initial weight of each freeze‐dried hydrogel sample (Wi) was recorded, and the samples were immersed in phosphate‐buffered saline (PBS, pH 7.4) at 37°C. At predetermined time intervals, the hydrogels were removed from the medium, gently blotted with filter paper to remove excess surface moisture, and weighed (W_f_). The swelling ratio (%) was calculated using Equation (2):

(2) $$\mathrm{Swelling}\;\mathrm{ratio}=\frac{Wf}{Wi}\;X\;100$$

To determine the degradation behavior of the hydrogel system, 1 mL of each hydrogel formulation was prepared, and the initial wet weight was recorded as W_Day 0_. The samples were incubated in PBS (pH 7.4) at 37°C for predetermined time points. At each interval, the samples were removed, gently blotted to remove surface liquid, and reweighed W_Day N_. The percentage degradation was calculated using Equation (3):

(3) $$\mathrm{Degradation}\;\mathrm{ratio}(\%)=W_{\mathrm{Day}\;0}\;-\frac{W_{\mathrm{Day}\;N}}{W_{\mathrm{Day}\;0}}\;X\;100$$

All experiments were conducted in (*n* = 3) triplicate for each hydrogel formulation, and results are reported as mean ± standard deviation.

#### 2.2.15 In‐Vitro Drug loading and Release Into Cl‐Plu‐Cl/PDMA/PEI(GA) Hydrogel

Amoxicillin was loaded into the Cl‐Plu‐Cl/PDMA/PEI(GA) hydrogel by dissolving the required amount of amoxicillin powder directly into cold PBS (pH 7.4) to achieve the desired feed concentration. Lyophilized Cl‐Plu‐Cl, PDMA, and PEI(GA) were then added sequentially under gentle stirring at 4°C–8°C until completely dissolved. The final polymer solution was adjusted to maintain a total polymer content of 20% (w/v) with PEI(GA) at 0.01% (w/v). The prepolymer solution was incubated at 37°C to induce a sol‐gel transition, forming a fully cross‐linked, solid hydrogel with no residual free liquid phase. Therefore, all amoxicillin was assumed to be physically entrapped within the hydrogel network. The actual drug content was confirmed indirectly by performing an in vitro release study, and the loading efficiency (LE%) was calculated using Equation (4):

(4) $$\mathrm{Loading}\;\mathrm{Efficiency}\;\left(\mathrm{LE}\right)\%=\frac{W_{release}}{W_{initial}}\;X\;100$$

where W_released drug_ is the total amount of amoxicillin released over the study period, and W_initial drug_ is the initial amount of drug added during hydrogel preparation. All loading experiments were performed in triplicate, and results are reported as mean ± standard deviation.

The in vitro release profile of amoxicillin from the Cl‐Plu‐Cl/PDMA/PEI(GA) hydrogel was evaluated using a dialysis bag diffusion method. Briefly, 2 mL of each of the fully crosslinked amoxicillin‐loaded hydrogels was transferred into a pre‐swollen dialysis bag (molecular weight cut‐off: 8–14 kDa). The dialysis bag was immersed in 20 mL of phosphate‐buffered saline (PBS) at two pH conditions (7.4 and 5.0) to simulate physiological and mildly acidic microenvironments, respectively. The release medium was maintained at 37°C with sink condition throughout the study. At predetermined time intervals, one mL aliquots of the release medium were withdrawn and immediately replaced with an equal volume of fresh, pre‐warmed buffer to maintain sink conditions. The amount of amoxicillin released into the medium was quantified by measuring absorbance at 230 nm with a UV–vis spectrophotometer, and the concentration was determined from a standard calibration curve prepared under the same conditions (Figure S3). The cumulative percentage of amoxicillin released was calculated using Equation (5):

(5) $$\mathrm{Cumulative}\;\mathrm{release}\;(\%)=\frac{W_{release\;at\;time\;\left(t\right)}}{W_{initial\;at\;time\;\left(to\right)}}\;X\;100$$

where W_released at time t_ is the total amount of drug released at each time point, and W_initial loaded_ is the initial amount of drug incorporated into the hydrogel.

All release experiments were performed in triplicate for each pH condition, and results are presented as mean ± standard deviation. The release profiles were further analyzed by fitting the data to standard kinetic models (e.g., Higuchi, Korsmeyer‐Peppas) to evaluate the mechanism of drug release from the cationic hydrogel network.

#### 2.2.16 In Vitro Antibacterial Activity of Amoxicillin‐Loaded Hydrogels

The antibacterial effectiveness of amoxicillin‐loaded Cl‐Plu‐Cl/PDMA/PEI(GA) hydrogels was tested against *S. aureus* and methicillin‐resistant *S*. *aureus* (MRSA) using a suspension killing assay and an agar diffusion (zone of inhibition) test, using a previously reported method with few modifications as detailed in the Supplementary Information (page S13) [19, 39]. Briefly, drug‐loaded and blank hydrogels were incubated with standard bacterial suspensions at 37°C, and the surviving bacteria were measured as log CFU mL^−^^1^ and the diameter of the inhibition zones on nutrient agar plates to evaluate antibacterial performance.

#### 2.2.17 Hemolysis Activity Assay

The hemolytic activity of the Cl‐Plu‐Cl/PDMA/PEI(GA) hydrogels was evaluated according to a previously reported protocol [16], Red blood cells (RBCs) were obtained from defibrinated sheep blood, which was centrifuged at 500 × g for 10 min. The RBCs were then washed three times with PBS to eliminate plasma proteins. The washed RBCs were diluted to 10% v/v in saline and aliquoted into separate vials (500 µL each). To each vial, 100 µL of the hydrogel or fibrin gel sample was added, and the mixture was incubated at 37°C. Saline solution and 0.1% Triton X‐100 were used as the negative and positive controls, respectively. After incubation for 3 h, the samples were centrifuged at 500 × g for 10 min. The supernatants were then transferred to a new 96‐well plate, and the absorbance of the released hemoglobin was measured at 540 nm using a microplate reader. The hemolysis ratio (%) was calculated using the following Equation (6):

(6) $$Hemolysis\;Ratio\;\left(\%\right)=\frac{OD_{sample}-OD_{negative}}{OD_{positive}-OD_{negative}}\;X100$$

where OD_sample_, OD_negative_, and OD_positive_ represent the absorbance values of the sample, negative control (saline), and positive control (0.1 % Triton X‐100), respectively.The procedures used to assess the hemolytic activity of hydrogel degradation products collected after incubation under physiological conditions are described above. All hemolysis tests were conducted in triplicate, and results are expressed as mean ± standard deviation.

#### 2.2.18 In Vitro Blood Clotting and Clotting Time Assay

Heparinized whole sheep blood was used to assess the coagulation performance of the Cl‐Plu‐Cl/PDMA/PEI(GA) hydrogels. In the vial inversion test, a defined volume of prepolymer solution was mixed with whole blood in a glass vial and incubated at 37°C for a set time to allow in situ gelation, after which the vial was gently inverted, and the extent of blood retention within the hydrogel and any flowing, uncoagulated blood were visually recorded and photographed. The blood clotting ability of the Cl‐Plu‐Cl/PDMA/PEI(GA) hydrogels was further evaluated using a standard clotting time assay, according to a previously reported protocol [40], with minor modifications. Briefly, 100 µL of each hydrogel formulation and control was used to coat the bottom of individual wells in a 24‐well plate. Fresh citrated rat blood was mixed with a 0.1 M calcium chloride solution at a 10:1 (v/v) ratio and vortexed for 10 s to initiate coagulation. Subsequently, 50 µL of the recalcified blood sample was carefully added to each coated well. At predetermined time points, each well was gently washed with PBS to remove any uncoagulated blood components, and the time required for a stable, firm clot to form in each well was recorded as the clotting time. All measurements were performed in triplicate, and results are reported as mean ± standard deviation.

### 2.3 In‐vivo Haemostasis

An in vivo mouse liver bleeding model was employed to assess the hemostatic performance of the developed hydrogels, following a previously established protocol [41]. Male ICR mice (10–24 g; Central Animal Facility, Department of Materials Engineering, Indian Institute of Science, Bangalore, India) were used for all experiments. All animal procedures were performed in accordance with the guidelines approved by the Institutional Animal Ethics Committee (IAEC) of the Central Animal Facility, Indian Institute of Science, Bangalore, India (CPCSEA Registration No. CAF/Ethics/147/2025, dated 09/05/2025). Briefly, mice were anesthetized with isoflurane inhalation and subjected to a midline laparotomy. After carefully removing the serous fluid surrounding the liver, a pre‐weighed filter paper (Whatman Grade 3, 90 mm diameter) with an initial weight (Wp_0_) was placed beneath the liver on a parafilm sheet (2‐inch; Bemis Co. Inc., Neenah, WI, USA). Each mouse was positioned at a 45° incline to facilitate consistent blood collection. A standardized 5 mm incision was then made at the center of the left liver lobe using a sterile surgical scalpel. Immediately after the incision, the Cl‐Plu‐Cl/PDMA/PEI(GA) hydrogel prepolymer solution was applied directly to the bleeding site using a syringe, without cauterization. After 3 min, the filter paper was collected and weighed (Wp), and the total blood loss was calculated as (Wp−Wp_0_). Each condition was tested in six independent animals (*n* = 6), and the results were expressed as mean ± standard deviation to ensure statistical robustness. Two control groups were included: (i) a positive control treated with the commercial hemostatic adhesive Truseal, and (ii) a negative control in which no material was applied.

#### 2.3.1 Statistical Analysis

One‐way analysis of variance (ANOVA) followed by Tukey's post hoc test was performed to compare the adhesion score, adhesion extent, and hemostatic performance among the experimental groups. A *p*‐value of < 0.05 was considered statistically significant. All statistical analyses were conducted using OriginPro software (OriginLab Corporation, Northampton, MA, USA).

## 3 Results and Discussion

### 3.1 Synthesis and Characterization of the Hydrogels' Prepolymers

**FIGURE 2:**
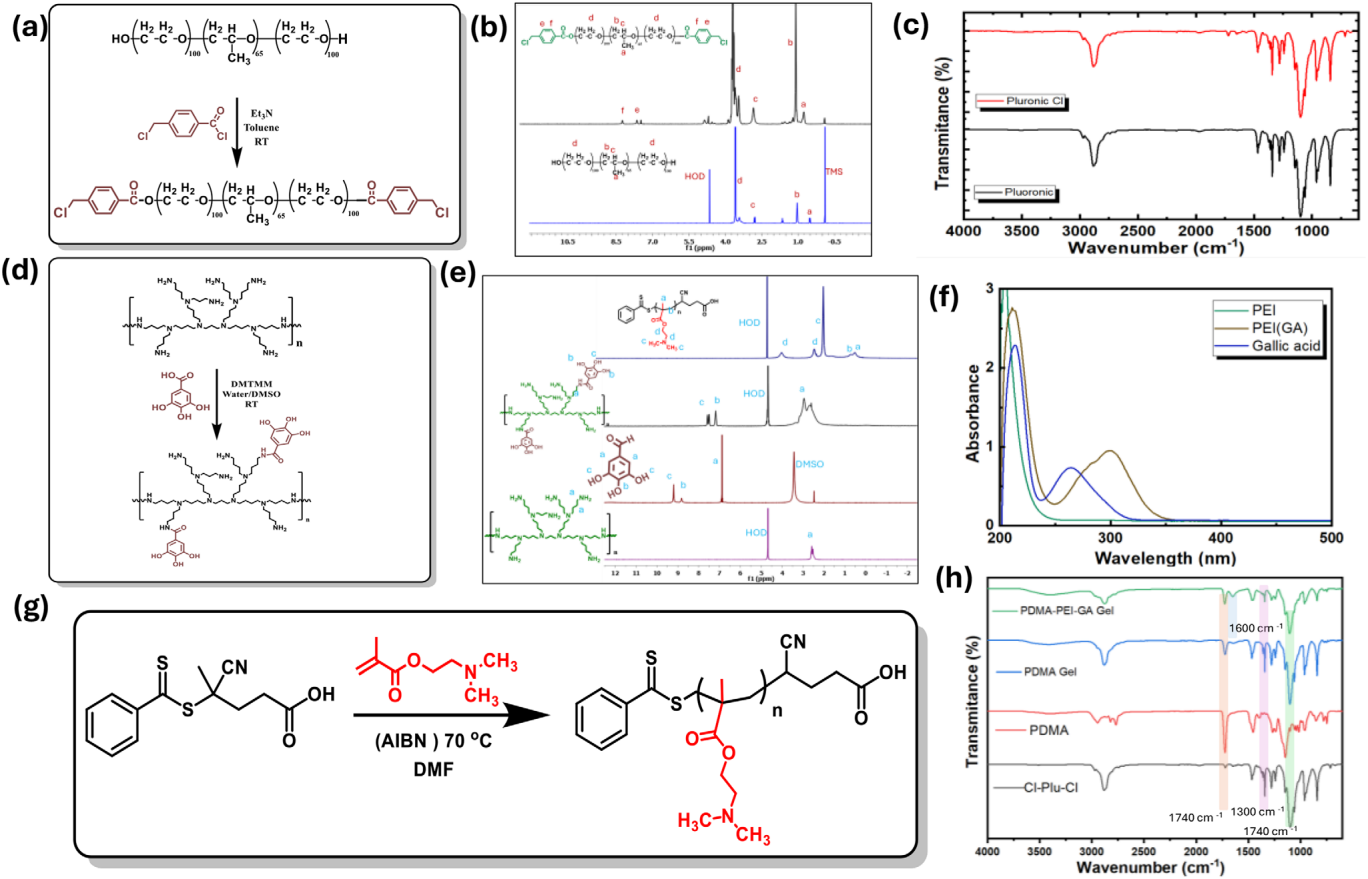
Chemical synthesis and structural characterization of the Cl‐Plu‐Cl/PDMA/PEI(GA) hydrogel system. (a) Schematic representation of the synthetic route for halide‐terminated Pluronic (Cl‐Plu‐Cl) via esterification. (b) Representative ^1^H NMR spectra of Pluronic and Cl‐Plu‐Cl confirming successful end‐group functionalization, showing characteristic aromatic benzoyl chloride peaks and shifts in PPO/PEO block signals. (c) FT‐IR spectra of Pluronic and Cl‐Plu‐Cl demonstrating the appearance of ester carbonyl bands after benzoyl chloride modification. (d) Schematic representation of PEI(GA) synthesis via DMTMM‐mediated coupling of gallic acid to branched polyethyleneimine. (e) ^1^H NMR spectra of PEI, gallic acid, and PEI(GA) verifying successful GA conjugation through the appearance of aromatic and phenolic proton signals. (f) UV–vis absorption spectra of free PEI, PEI(GA), and gallic acid, showing characteristic peak shifts upon GA grafting onto PEI. (g) Schematic of RAFT polymerization used to prepare the PDMA macro‐RAFT agent. (h) FT‐IR spectra of Cl‐Plu‐Cl, PDMA, PDMA hydrogel, and Cl‐Plu‐Cl/PDMA/PEI(GA) hydrogel, highlighting ester carbonyl stretching (∼1740 cm^−^^1^), imine/amine bands (∼1600 cm^−^^1^), and C–N/C–O stretching (∼1300 cm^−^^1^), confirming successful crosslinking and PEI(GA) incorporation.

To develop a multifunctional, pH‐responsive injectable hydrogel system, we first functionalized Pluronic F‐127 to introduce reactive sites at both termini. Chloromethyl‐terminated Pluronic (Cl‐Plu‐Cl) was synthesized via a targeted esterification reaction, in which the terminal hydroxyl groups of F‐127 were reacted with 4‐(chloromethyl)benzoyl chloride in anhydrous toluene, using triethylamine as both a base and HCl scavenger (Figure 2a). This reaction proceeded smoothly, forming stable ester linkages while neutralizing the generated HCl in situ. The resulting Cl‐Plu‐Cl serves as a reactive site for subsequent crosslinking.

Successful incorporation of chloromethylbenzoyl units at both ends of the Pluronic F127 backbone was confirmed by ^1^H NMR and FT‐IR spectroscopy. In the ^1^H NMR spectrum, characteristic aromatic signals appeared between 7.5–8.0 ppm, confirming the conjugation of the 4‐chloromethyl benzoyl groups to the Pluronic backbone (Figure 2b). FT‐IR spectra further supported this modification, showing a strong ester carbonyl stretching band at ∼1735 cm^−^^1^ and new aromatic C─H bending vibrations around 750 cm^−^^1^, validating the structural modification(Figure 2c). From the integral ratio of aromatic benzoyl protons to PPO/PEO methylene signals, the degree of chloromethylbenzoyl functionalization of Pluronic F‐127 was estimated to be ∼99%–100% per chain, confirming nearly complete end‐group modification of the crosslinker. This bifunctional Pluronic derivative provides a robust and versatile platform for building amphiphilic, thermoresponsive hydrogel networks tailored for biomedical applications [42]. Another component of hydrogels is gallic acid‐functionalized polyethyleneimine (PEI(GA), which was prepared by conjugating gallic acid to the primary amine groups of branched PEI via DMTMM‐mediated carbodiimide coupling (Figure 2d) [43]. Successful conjugation was evidenced by ^1^H NMR, with aromatic proton signals appearing at 6.8–7.2 ppm and amide protons at ∼8.2 ppm, alongside PEI backbone peaks around 2.5–3.0 ppm (Figure 2e). FT‐IR spectra showed new amide C═O stretching (∼1650 cm^−^^1^) and aromatic C═C vibrations (∼1510 cm^−^^1^), while the broad N─H stretching of PEI appeared at ∼3300–3400 cm^−^^1^ (Figure 2h). UV–vis spectra of PEI‐GA exhibited a characteristic absorption peak at ∼280–300 nm, confirming the presence of gallic acid's aromatic moiety and successful conjugation (Figure 2f) [44, 45]. Quantitative ^1^H NMR analysis indicated a gallic‐acid substitution degree of approximately 21% of primary amines, calculated from the ratio of aromatic GA signals (6.8–7.2 ppm) to PEI methylene protons, in good agreement with the degree of substitution estimated from the GA calibration curve by UV–vis spectroscopy (19.4% substitution).

For the hydrogel crosslinking network, poly(N, N‐dimethylaminoethyl methacrylate) (PDMA) was synthesized via RAFT polymerization to achieve precise control over molecular weight and polymer architecture (Figure 2b) [46]. The crosslinking functionality of PDMA is attributed to its tertiary amine groups, which serve as reactive sites for network formation [47]. The successful RAFT polymerization of PDMA was confirmed by ^1^H NMR, showing characteristic peaks for –N(CH_3_)_2_ at ∼2.2–2.3 ppm and –CH_2_–N at ∼2.5–3.0 ppm, with the absence of vinyl signals indicating complete monomer conversion. FT‐IR spectra further supported this, displaying strong C═O stretching near 1725 cm^−^^1^, N─H stretching around 3400 cm^−^^1^, and C─N stretching at ∼1140 cm^−^^1^, confirming the presence of amide linkages and tertiary amines (Figure 2h) [46]. Overall, these results confirm the successful synthesis of the three hydrogel prepolymer components Cl‐Plu‐Cl, PDMA, and PEI(GA), providing a modular and versatile system for constructing thermoresponsive, multifunctional injectable hydrogels with potential applications in hemostasis and wound healing.

### 3.2 Dually Crosslinked pH‐Responsive and Thermoresponsive Injectable Hydrogels: In Situ Gelation from Reactive Prepolymers

**FIGURE 3:**
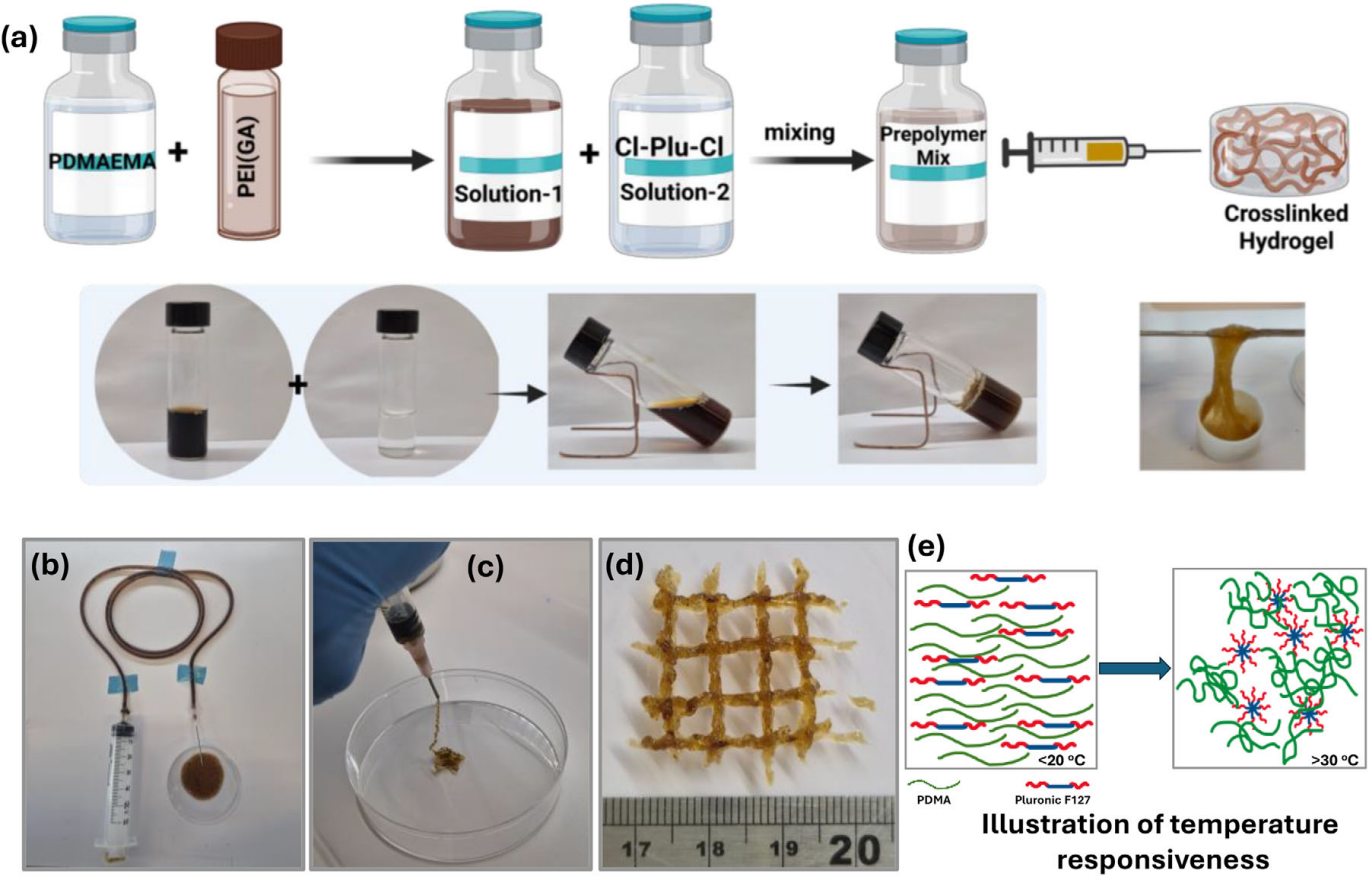
(a) Schematic illustration of Cl‐Plu‐Cl/PDM/PEI(GA) hydrogel preparation showing sequential mixing of polymeric precursors to form a crosslinked hydrogel network. Final appearance of the fully crosslinked hydrogel, exhibiting robust elasticity and cohesive network formation. (b) The injectability of the prepolymer solution before crosslinking through a long silicone tube demonstrates excellent flowability. (c) Injectability of the viscous, semi‐solid pre‐gel phase through a 21G needle, validating its suitability for minimally invasive delivery. (d) Manually printed a hydrogel scaffold through a 21G syringe‐needle, confirming printability and structural integrity. (e) illustration of the temperature responsiveness of both PDMA and Pluronic F127.

Injection of an aqueous mixture of PDMA (Mn = 27,000 g/mol, PDI = 1.30), Cl‐Plu‐Cl (Mn = 12,600 g/mol), and PEI(GA) (Mn = 60,000 g/mol, DS = 27%) into PBS (pH 7.4) resulted in the formation of chemically crosslinked hydrogels (Figure 3a). The gelation time, ranging from approximately 1 to 4 min at 37°C, was influenced by the PDMA‐to‐Cl‐Plu‐Cl ratio and the prepolymer concentration (w/w). The tunable gelation of our hydrogels is primarily driven by the nucleophilic substitution reaction between the tertiary amines of PDMA and PEI(GA) with the activated chloride termini of Cl‐Plu‐Cl under physiological conditions [32, 47]. A key advantage of our hydrogel system is the amphiphilic and thermoresponsive properties of both PDMA and Pluronic F‐127. Upon injection at human physiological temperature (∼37°C), the Pluronic F‐127 segments undergo temperature‐induced micellization and partial physical crosslinking, thereby concentrating the reactive groups locally and accelerating the chemical gelation reaction with PDMA and PEI(GA). At the same time, PDMA exhibits mild temperature responsiveness, increasing the mobility of its polymer chains at physiological temperatures and facilitating network formation. This combined thermoresponsive behavior ensures rapid in situ gelation, even with minimally invasive delivery, while maintaining injectability before crosslinking.

The reactive tertiary amine groups in PDMA promote rapid chemical crosslinking without producing cytotoxic by‐products such as quaternary ammonium derivatives, as verified through model gelation studies. Notably, no gelation occurred when PDMA was combined with unmodified Pluronic F‐127, underscoring the essential role of the Cl‐Plu‐Cl modification in enabling covalent network formation. The gelation kinetics of the system are highly tunable, allowing the prepolymer solutions to be conveniently delivered through long, narrow tubing before complete gelation. The injectable prepolymer solution also exhibits excellent printability and can be utilized as a 3D printing ink, as demonstrated by the manual printing of a representative structure. The workable printing time after mixing the prepolymers ranges from 2 to 10 min (at 16% w/v and 25°C), depending on polymer concentration and temperature. Both factors govern the available delivery window at higher temperatures accelerate gelation, while lower temperatures extend it, thereby enabling precise spatiotemporal control over hydrogel formation for in vivo applications (Figure 3a–e; Movie S1 and Movie S2).

**FIGURE 4:**
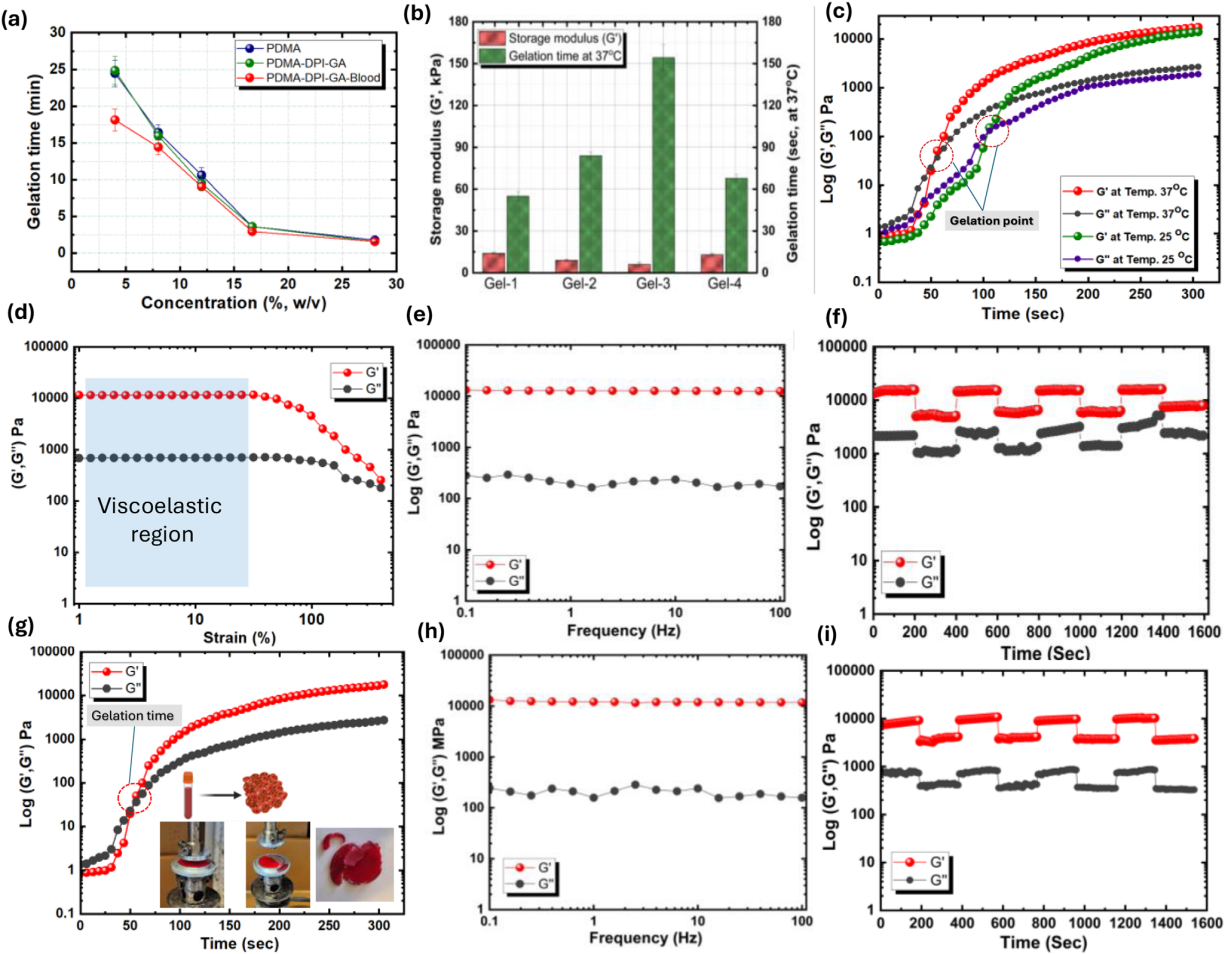
(a) Gelation time vs. polymer concentration for Gel‐1 at 37°C, determined by oscillatory rheology. (b) Variation in gelation time and storage modulus (G') for Gel‐1, Gel‐2, Gel‐3, and Gel‐4 at 37°C. (c) Temperature‐dependent gelation kinetics of Gel‐1, illustrating differences in gelation behavior at 25°C and 37°C. (d) Strain‐sweep analysis of Gel‐4 at ω = 6.3 rad s^−^^1^ showing storage (G′) and loss (G″) moduli as a function of strain and defining the linear viscoelastic region. (e) Frequency‐sweep analysis of Gel‐1 at γ = 5%, showing the dependence of G' and G" on angular frequency. (f) Alternating strain‐sweep test on freshly prepared Gel‐1, subjected to low (0.1%) and high (200%) strains for 2 min per step at ω = 6.3 rad s^−^^1^, demonstrating reversible disruption and recovery of the network. (g) Effect of whole‐blood contact on the gelation time of Gel‐1. (h) Frequency‐sweep curves of Gel‐1 formed in the presence of whole blood at γ = 5%, indicating the stability of the blood–hydrogel composite. (i) Alternating strain‐sweep test of Gel‐1 in whole blood under low (0.1%) and high (200%) strains for 2 min per step at ω = 6.3 rad s^−^^1^, showing its dynamic mechanical response under repetitive deformation.

Characterization of the resulting amphiphilic and stimuli‐responsive hydrogels via SEM, FT‐IR spectroscopy, gelation, swelling, degradation, and rheology confirmed the formation of a robust network. Gelation times were further evaluated by varying prepolymer concentration (Figure 4a), and the influence of the PDMA:Cl‐Plu‐Cl ratio on hydrogel formation is summarized in Table 1. Injection of a 1 g prepolymer mixture (4:6:0.01 Cl‐Plu‐Cl:PDMA: PEI(GA)) resulted in an experimentally observed temperature increase of only ∼1 °C, well within safe limits for in situ biomedical applications [47]. Efficient gelation occurred even slightly below physiological temperatures, demonstrating that the thermoresponsive synergy between PDMA and Pluronic F‐127 provides a self‐accelerating gelation mechanism upon contact with body tissues (Figure 3e). Sol fractions ranged from 2%–6% (w/w) in DMF and 1–5% (w/w) in PBS (pH 7.4) at 37°C, indicating minimal unreacted components. The hydrogels maintained structural integrity over one week and remained stable between 0°C and >70°C in PBS and under basic conditions (2 N KOH). Collectively, the thermoresponsive nature of both PDMA and Pluronic F‐127 facilitates minimally invasive injection and simultaneously triggers rapid, localized gelation at body temperature. This accelerates network formation and enhances hydrogel retention at the wound site, a critical feature for in situ hemostatic applications where immediate sealing and strong tissue adhesion are essential to prevent blood loss.

### 3.3 Injectability Evaluation of Prepolymer Solutions

Injectability is a critical parameter for minimally invasive hemostatic materials, particularly for delivery through narrow catheters or endoscopic devices [48, 49]. To mimic clinical conditions, hydrogel precursor solutions were injected via 21 and 23G needles connected to narrow medical tubing (1.2 mm inner diameter, 90 cm length), which represented catheter lumens. Manual injection at flow rates of 1.0–1.5 mL/min required extrusion forces of 2–4 N, well within feasible clinical limits. The solutions exhibited smooth, continuous flow without clogging, backflow, or phase separation (Figure 3b,c) and in Movies S3 and S4. Rheological analysis revealed moderate viscosity (∼250–400 mPa s) with pronounced shear‐thinning behavior, which facilitates injection through narrow lumens and enhances retention at the target site by reducing post‐injection flow [50]. No air bubbles or precipitation were observed, confirming the stability of the precursor solutions during handling. Collectively, these findings indicate that the developed hydrogels possess excellent injectability and handling properties, supporting their potential for rapid, precise, and minimally invasive hemostatic application in dynamic bleeding scenarios.

### 3.4 Rheological Properties of Cl‐Plu‐Cl/PDMA/PEI(GA) Gels

Injectable hydrogels designed for hemostatic applications must combine excellent injectability, shear responsiveness, tunable gelation, and robust mechanical performance. The Cl‐ Plu‐ Cl/PDMA/PEI(GA) prepolymer solution exhibited pronounced shear‐ thinning behavior, confirmed by successful injection through a 1. 1.5 m tube and a 21‐ gauge needle. Before gelation at 25°C and 37°C flow curves showed a significant decrease in viscosity with increasing shear rate, characteristic of non‐Newtonian fluids (Figure S4). This shear‐thinning response results from electrostatic interactions, π–π stacking among gallic acid groups, and hydrophobic associations within the dual‐network system. Cl‐Plu‐Cl/PDMA blends demonstrated stronger shear‐thinning than PDMA alone, highlighting the synergistic effect of both networks. Rheological analysis of the precursor solution before gelation revealed comparable storage (G′) and loss (G″) moduli, indicating a mainly viscous nature, while viscosity gradually increased as gelation progressed. At room temperature, the precursor remained injectable for about 7–8 min, depending on the polymer concentration and the Cl‐Plu‐Cl:PDMA ratio (Figure 4a). Gelation kinetics were systematically studied for four formulations (Gel‐ 1 to Gel‐ 4). Gel‐ 1 showed the shortest gelation time and the highest storage modulus, indicating rapid and efficient network formation (Figure 4b,c) and (Figure S5 a‐c). Gelation time decreased as polymer concentration increased, from 24. 5 min at 4% to 1. 1.8 min at 28% (w/v), reflecting optimized chloride‐ to ‐amine ratios (0.89 mm/0.005 mm for Gel‐ 1, 1. 1.0 mm/0.0031 mm for Gel‐ 2, 1. 1.1 mm/0.0016 mm for Gel‐ 3, and 0.89 mm/0.005 mm for Gel‐ 4). Across all formulations, faster‐gelling hydrogels generally had higher mechanical strength: Gel‐1, Gel‐2, Gel‐3, and Gel‐4 gelled in 54, 82, 155, and 68 s, with corresponding storage moduli of 14.3, 10, 7, and 14 kPa (Figure 4b). Gelation was strongly affected by temperature, with Gel‐1 forming a solid network in 54 s at 37°C, compared with 106 s at 25°C, illustrating the thermoresponsive behavior of PDMA and Pluronic F 127 (Figure 4c).

The linear viscoelastic region (LVR) of the hydrogels was identified as the strain range over which the network retains its structural integrity. As shown in (Figure 4d), G′ stayed constant from 0.1% to 60% strain, establishing a wide operational window for rheological measurements and ensuring predictable mechanical behavior under physiological shear. Within this LVR, frequency sweeps (0.1–100 Hz) indicated a dominant storage modulus (G′ > G″) across all hydrogels, confirming their mainly elastic nature (Figure 4e; Figure S6a–c). Strain sweep tests (1%–200%) showed a sharp drop in G' approaching G″ at higher strains, pinpointing the yield point and demonstrating network stability under stress (Figure 4f; Figure S6d,e). Gelation in the presence of whole blood occurred slightly faster (∼51 s at 37°C) than in PBS (Figure 4g), likely due to electrostatic interactions between cationic precursors and negatively charged blood components, which promote localized aggregation and additional non‐covalent crosslinking. Despite this slight acceleration, the hydrogels formed strong networks with mechanical properties similar to those in PBS, highlighting the system's resilience under physiological conditions. Stability in whole blood was further confirmed by frequency sweep tests (Figure 4h) and alternating strain sweeps (Figure 4i), which showed that the network responds dynamically to low (0.1%) and high (200%) strains while maintaining recoverability.

Cyclic strain tests demonstrated the self‐healing and recoverable nature of the hydrogel networks. At low strain (*γ*  =  1 %), the gels exhibited solid‐like behavior (G'/ G“), while at high strain (*γ*  =  200 %), they transiently decreased (G' /G”) ratio than (*γ*  =  1 %), indicating reversible sol‐gel transitions (Figure 4f,i). Upon returning to low strain, both moduli fully recovered over multiple cycles, confirming the hydrogel's ability to regenerate its structure after mechanical disruption, a key requirement for smooth injection through surgical needles. Mechanical strength and viscoelastic performance were strongly influenced by formulation.

Gels with higher Cl^−^: amine ratios exhibited increased G' and G“, reflecting enhanced crosslinking density and network stiffness. Lower G”/G' ratios indicated reduced viscous dissipation, promoting improved wettability and potential tissue adhesion, essential for effective hemostasis. Gel‐1 and Gel‐4 showed the highest G' values; however, Gel‐4, lacking PEI(GA), exhibited slightly lower modulus due to the absence of tertiary amines that facilitate additional covalent and *π*–*π* interactions. Strain‐sweep data confirmed that dynamic physical interactions, such as hydrogen bonding, *π*–*π* stacking, and electrostatic associations, dominate network behavior, providing flexibility, recoverability, and shear responsiveness. Overall, the Cl‐Plu‐Cl/PDMA/PEI(GA) hydrogels integrate shear‐thinning injectability, rapid and tunable gelation, robust mechanical strength, and reversible sol‐gel transitions within a wide LVR (0.1%–60%). This combination ensures smooth delivery, immediate recovery of network integrity, and mechanical resilience under physiological and surgical shear stresses. The ability of the hydrogels to form and regenerate a robust network under varying strain and in the presence of blood highlights their multifunctional potential for minimally invasive hemostatic and regenerative applications, where both ease of administration and structural performance are critical. These rheological trends align with the underlying nucleophilic substitution between tertiary amines on PDMA and the chloromethyl benzoyl chloride end groups of Cl‐Plu‐Cl, which results in a densely crosslinked amphiphilic network at physiological pH without external initiators. Increasing Cl‐Plu‐Cl content enhances the effective crosslink density, thereby raising G' and the compressive modulus. At the same time, the flexible PEO/PPO backbone and dynamic ionic interactions provide shear‐thinning and self‐healing properties by enabling reversible disruption and reformation of physical junctions under load.

### 3.5 In Vitro Swelling, Degradation, and Microstructure Characterization of the Hydrogels

**FIGURE 5:**
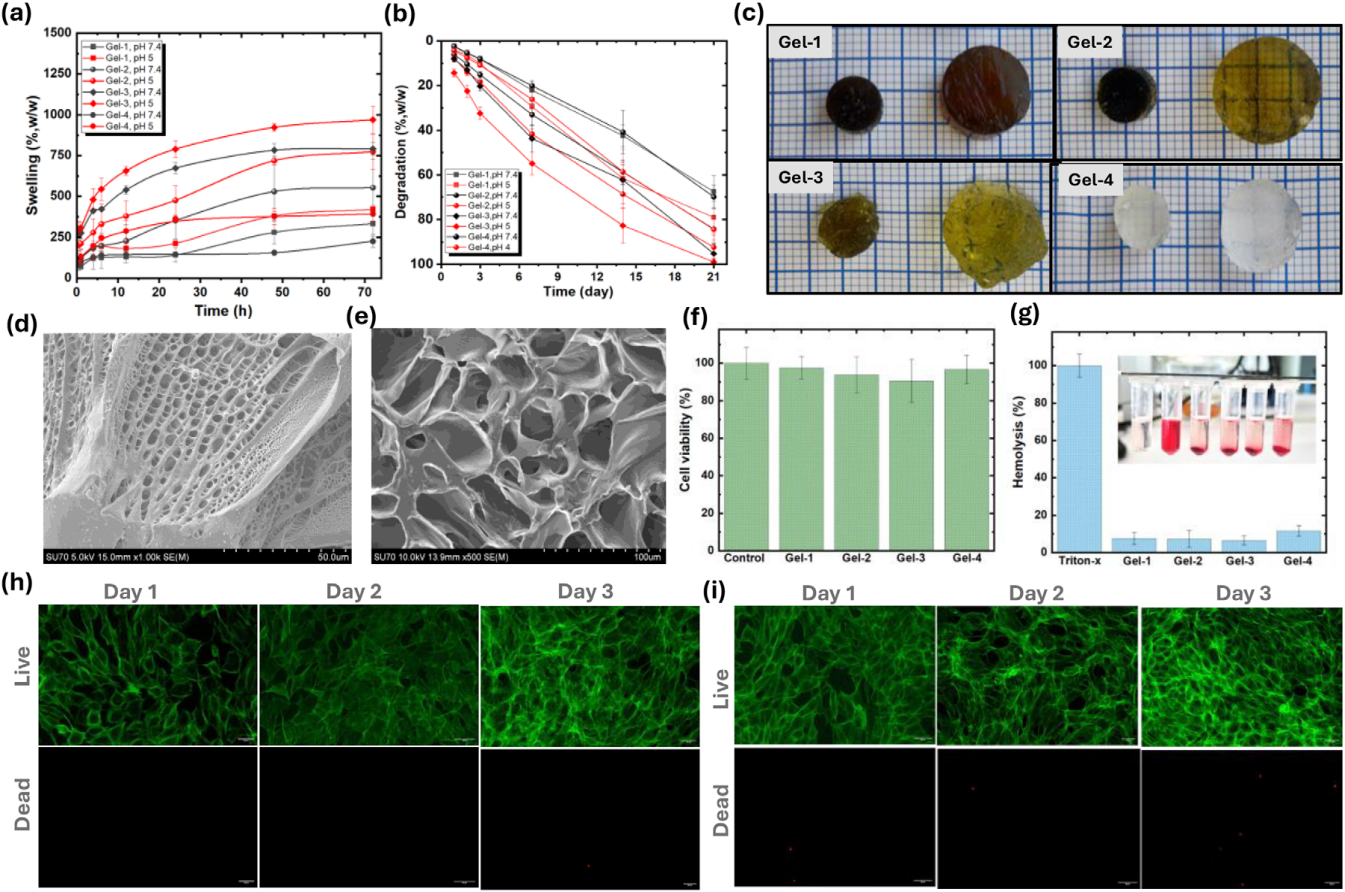
(a) Swelling behavior of Gel‐1 to Gel‐4 under physiological (pH 7.4) and acidic (pH 5.0) conditions, showing pronounced pH‐responsive swelling, particularly for Gel‐1 and Gel‐2. (b) In vitro degradation profiles of Gel‐1 to Gel‐4 at pH 7.4 and 5.0 over 21 days, demonstrating accelerated mass loss under acidic conditions, most notably in Gel‐3 and Gel‐4. (c) Digital images of Gel‐1 to Gel‐4 before and after equilibrium swelling, highlighting substantial dimensional expansion and water uptake across formulations. (d) SEM surface morphology of Gel‐1, showing a porous, interconnected network. (e) Cross‐sectional SEM image of Gel‐1, revealing a sponge‐like internal architecture. (f) Cell viability of L929 fibroblasts after 24 h incubation with PDMA/PEI(GA)/Cl‐Plu‐Cl hydrogels (Gel‐1 to Gel‐4), indicating excellent cytocompatibility relative to untreated controls. (g) Hemolysis percentage for Gel‐1 to Gel‐4, confirming hemocompatibility; Triton X serves as the positive control, and the inset shows corresponding tube images with negligible red blood cell lysis for all hydrogel samples. (h) Live/dead fluorescence images of NIH 3T3 cells in the control group without hydrogel exposure over 3 days. (i) Live/dead fluorescence images of NIH 3T3 cells cultured in the presence of representative Gel‐1 over 3 days, demonstrating predominantly live cells and minimal cytotoxicity (scale bar = 50 µm).

The rapid degradation of hemostats in the body after hemostasis is crucial to prevent them from lingering and causing undesired side effects, and to facilitate wound healing. As a preliminary study to investigate in vitro biodegradability, the swelling and degradation kinetics of Cl‐Plu‐Cl/PDMA/PEI(GA) gels in PBS at 37°C were analyzed. As shown in (Figure 5a,b), Cl‐Plu‐Cl/PDMA/PEI(GA) gels with a higher Cl^−^ to tertiary amine ratio exhibited a higher swelling rate initially and a faster degradation rate thereafter. This could be attributed to the water retention and hydrolysis of the ester linkage of Cl‐Plu‐Cl [33]. It took approximately 3–4 weeks for all Cl‐Plu‐Cl/PDMA/PEI(GA) gels to degrade completely. This result suggests that the Cl‐Plu‐Cl/PDMA/PEI(GA) gel could rapidly degrade in the body after hemostasis is completed in the targeted tissue. The swelling characteristics of hydrogels play a pivotal role in absorbing wound exudate, promoting hemostasis, and facilitating tissue integration, thereby accelerating the tissue healing process [51, 52]. The swelling behavior of hydrogels with varying crosslinker concentrations is depicted in Figure 5b. The graph illustrates that all hydrogels initially exhibit rapid water absorption, with Gel‐4 demonstrating the highest rate. Subsequently, the rate of water absorption diminishes until reaching a state of swelling equilibrium. (Figure 5b) which shows the hydrogels after reaching swelling equilibrium, indicating that there is little volume difference among the different hydrogels. At equilibrium, the swelling ratios of Gel‐1, Gel‐2, Gel‐3, and Gel‐4 were determined to be 33.3 ± 0.75%, 55.4± 1.7%, 79.1 ± 0.38%, and 29.6 ± 0.37%, respectively (Figure 4a). Notably, the swelling rate of the Gel‐1 to Gel‐3 hydrogels declined because crosslinking density decreases.

Hydrogel degradation is also a critical parameter when hydrogels are applied in vivo. Because their clearance from the body depends on their degradation. In our hydrogels, degradation begins on the first day after equilibrium swelling and accelerates over time. In the Gel‐1, Gel‐2, Gel‐3, and Gel‐4 hydrogels, a higher degradation rate was observed at acidic pH. The Gel‐1, Gel‐2, Gel‐3, and Gel‐4 degraded 67.56 ± 7.15%, 84.33± 6.26, 95.23± 4.37 and 69.9±5.16 respectively (Figure 5b). The degradation mechanism of these hydrogels is as follows: we assume that the ester linkage of the Cl‐Plu‐Cl hydroxylase causes the hydrogels to swell significantly, leading to dissolution. The pH 5.0 degradation rate supports this hypothesis because, at lower pH, the free Tertiary amine is protonated, thereby accelerating hydrolysis of the ester linkage. The degradation ratios of Gel‐1, Gel‐2, Gel‐3, and Gel‐4 at pH 7.4 and pH 5.0 indicate that as crosslinking decreases, hydrogel degradation accelerates in Gel‐1, Gel‐2, and Gel‐3. To further investigate the influence of the Cl^−^ to tertiary amine ratio on the internal microstructure of the hydrogels, scanning electron microscopy (SEM) was performed on lyophilized Cl‐Plu‐Cl/PDMA/PEI(GA) gels with varying reactive species ratios (Figure 5d,e; Figure S7a–c). The SEM images revealed that gels with higher Cl^−^ to tertiary amine ratios exhibited a more continuous and densely connected polymer wall structure, indicative of a well‐formed, tightly crosslinked network. Conversely, hydrogels prepared with lower Cl^−^ to amine ratios exhibited a more porous and fibrous morphology, characterized by discontinuous polymer walls that resembled the microstructure typically observed in gelatin‐based systems [41]. These findings demonstrate that the hydrogel's internal architecture can be precisely tuned by adjusting the Cl^−^ to tertiary amine ratio, thereby modulating not only mechanical properties but also porosity and diffusion characteristics, which are essential for biomedical applications such as controlled drug delivery and cellular interactions in wound healing.

Cross‐sectional SEM images further confirmed that all hydrogels exhibited a porous, 3D network structure, which facilitates rapid water absorption (Figure 5d,e). Quantitative analysis revealed that the pore sizes for Gel‐1, Gel‐2, Gel‐3, and Gel‐4 were 37.01 ± 13.31 µm, 66.97 ± 12.32 µm, 82.80 ± 24.78 and 29.2 ±8.56 µm, respectively (Figure S7d). Notably, Gel‐1 exhibited significantly smaller pores compared to Gel‐3. This reduction in pore size is attributed to increased crosslinking between Cl‐Plu‐Cl, PDMA, and PEI(GA), as well as molecular chain entanglements facilitated by hydrogen bonding and *π*–*π* interactions. These interactions lead to a higher degree of crosslinking, resulting in the formation of smaller ice crystals during lyophilization and consequently smaller pore structures [53]. The porous and interconnected network of these hydrogels provides sufficient space for cellular growth, attachment, proliferation, and extracellular matrix secretion, underscoring their potential for regenerative medicine and tissue engineering applications [54].

### 3.6 Cytocompatibility and Hemocompatibility of the Hydrogels

The cytocompatibility of the developed hemostatic hydrogels was evaluated using the Alamar Blue assay in accordance with ISO 10993–5 guidelines (Figure 5f). Untreated L‐929 fibroblasts served as the baseline reference, and cells cultured in standard medium served as the negative control. The individual prepolymer components PDMA, PEI(GA), and Cl‐Plu‐Cl were tested at varying concentrations and showed minimal cytotoxicity toward L‐929 cells. The complete hydrogel formulations (Gel‐1 to Gel‐4) were subsequently assessed, and all groups showed normal cell morphology with viability ranging from 94% to 99%. A slight decrease in viability was observed only at higher concentrations of pure PDMA and PEI(GA), which could be attributed to changes in the culture medium or the high cationic charge affecting cellular activity. These results confirm that the crosslinked hydrogels display excellent cytocompatibility with negligible toxicity to mammalian cells. The mild cytotoxicity observed for free PDMA likely arises from its strong positive charge, which can interact with negatively charged cell membranes. In contrast, the hydrogels exhibited very low sol fraction values, indicating minimal release of unreacted PDMA, which explains the improved cell viability.

Hemocompatibility was examined through hemolysis testing (Figure 5g). According to the extent of hemoglobin release, materials causing less than 2% hemolysis are considered non‐hemolytic, those between 2% and 5% slightly hemolytic, and those above 5% hemolytic [55]. The prepolymers PDMA and PEI(GA) exhibited hemolysis rates of 5%–8%, indicating mild hemolytic activity. However, all hydrogel formulations showed lower hemolysis values, ranging from 2% to 5.5%, indicating they are slightly hemolytic but still within the acceptable range for biomedical use. Together, these hemolysis data suggest that the Cl‐Plu‐Cl/PDMA/PEI(GA) hydrogels exhibit acceptable in vitro hemocompatibility, with all formulations remaining about to 5% hemolysis threshold under the tested conditions. However, some compositions approach this limit at prolonged exposure. However, in vitro hemolysis alone cannot fully predict systemic blood compatibility; future work will therefore include detailed in vivo assessment of hematological parameters, coagulation indices, complement activation, and organ histology to comprehensively establish the clinical safety profile of these hydrogels. In line with these findings, live/dead fluorescence imaging of NIH 3T3 cells cultured with the hydrogels revealed predominantly calcein‐positive (live) cells and only sparse EthD‐III–positive (dead) cells over 3 days, visually corroborating their excellent cytocompatibility (Figure 5h,i). The reduction in hemolytic activity following hydrogel formation can be attributed to the crosslinked network, which limits direct polymer‐red blood cell contact and prevents the leaching of reactive components.

In addition, degradation products collected from hydrogels incubated under physiological conditions were characterized by FT‐IR and subjected to Alamar Blue and hemolysis assays, which showed no additional cytotoxic or hemolytic effects compared with the intact hydrogels (Figure S8a–c). Collectively, these findings demonstrate that the injectable hydrogels possess strong cytocompatibility and acceptable hemocompatibility, supporting their potential use as safe and effective materials for hemostatic and wound‐sealing applications. However, these in vitro assays and short‐term in vivo observations do not account for potential long‐term or systemic toxicity; thus, future research will focus on extended implantation studies with follow‐up over several weeks, including hematological analysis and histological examination of major organs and local tissues, to thoroughly establish the chronic safety profile of the hydrogels.

### 3.7 Radical Scavenging Activity

The UV–vis absorbance spectra (450–700 nm) and corresponding visual assessments of DPPH solutions incubated with various formulations revealed pronounced radical scavenging activity, particularly for the gel‐based systems, as evidenced by the characteristic decolorization from purple (unscavenged DPPH radical at ∼517 nm peak) to yellow (scavenged DPPH‐H). The DPPH assay revealed dose‐dependent radical scavenging activity in both synthesized polymers and hydrogels, with scavenging percentages quantified using a gallic acid calibration curve (Figure S9a), (*y* = 0.04786x + 0.07486, R^2^ = 0.9968) that correlated remaining DPPH absorbance at 517 nm with antioxidant concentration (0–100 µg mL^−^^1^). Under different DPPH concentrations of 25, 50, or 100 µm, when compared to those of crosslinked hydrogels 1–3 (50 mg mL^−^^1^), Hydrogel‐4 (50 mg mL^−^^1^) showed significantly higher radical scavenging activities (Figure S9b; 25 µm: 92.8%, 96.45%, 98.3% vs. 20.8%). All these results confirmed the antioxidative capability of injectable hydrogels with PEI(GA). The DPPH control (vial a, purple line) exhibited high absorbance (∼2.5–3.0) at 517 nm, confirming stable radical presence, while DPPH + PDMA (vial b, red line) showed minimal reduction (∼2.0–2.5 absorbance) with a persistent purple hue, indicating weak scavenging. In contrast, DPPH + Cl‐Plu‐Cl (vial c, pink line) and DPPH + PEI(GA) (vial d, green line) achieved strong activity with absorbance drops to ∼0.5–1.0 and clear yellow colors, comparable to the gallic acid positive control (vial e, blue dashed line, <0.5 absorbance). Among the gels, DPPH‐Gel‐1 (vial f, blue line) displayed moderate scavenging (∼1.0–1.5 absorbance at 517 nm) with remaining purple color, suggesting partial radical quenching, whereas DPPH‐Gel‐2 (vial g, black line), DPPH‐Gel‐3 (vial h, yellow line), and DPPH‐Gel‐4 (vial i, red line) demonstrated superior efficacy with near‐complete peak suppression (<0.3 absorbance) and full yellow decolorization, achieving >80%–90% reduction relative to the control (Figure S9b). These spectral and visual outcomes highlight the dose‐ or formulation‐dependent enhancement in antioxidant performance across Gel‐2 to Gel‐4. This enhancement is likely due to optimized matrix interactions that enable efficient electron transfer without byproduct interference or shifts beyond 517 nm, positioning these gels as promising scavengers for radical‐mediated applications (*n* = 3; mean ± SD) [37]. This behavior reflects the high density of phenolic hydroxyl groups on GA within the PEI(GA) segment, which can donate hydrogen atoms to neutralize DPPH radicals and form resonance‐stabilized phenoxy species, thereby granting the hydrogels an innate radical‐scavenging capacity. Although the DPPH assay confirms that incorporating PEI(GA) provides vigorous radical‐scavenging activity in vitro, these data do not directly demonstrate antioxidant effects in vivo; future studies will therefore focus on measuring ROS levels and oxidative‐stress markers in treated wound tissues to evaluate how the hydrogels' antioxidant activity influences healing outcomes under physiologically relevant conditions.

### 3.8 Excellent Bioadhesion of Hydrogels

**FIGURE 6:**
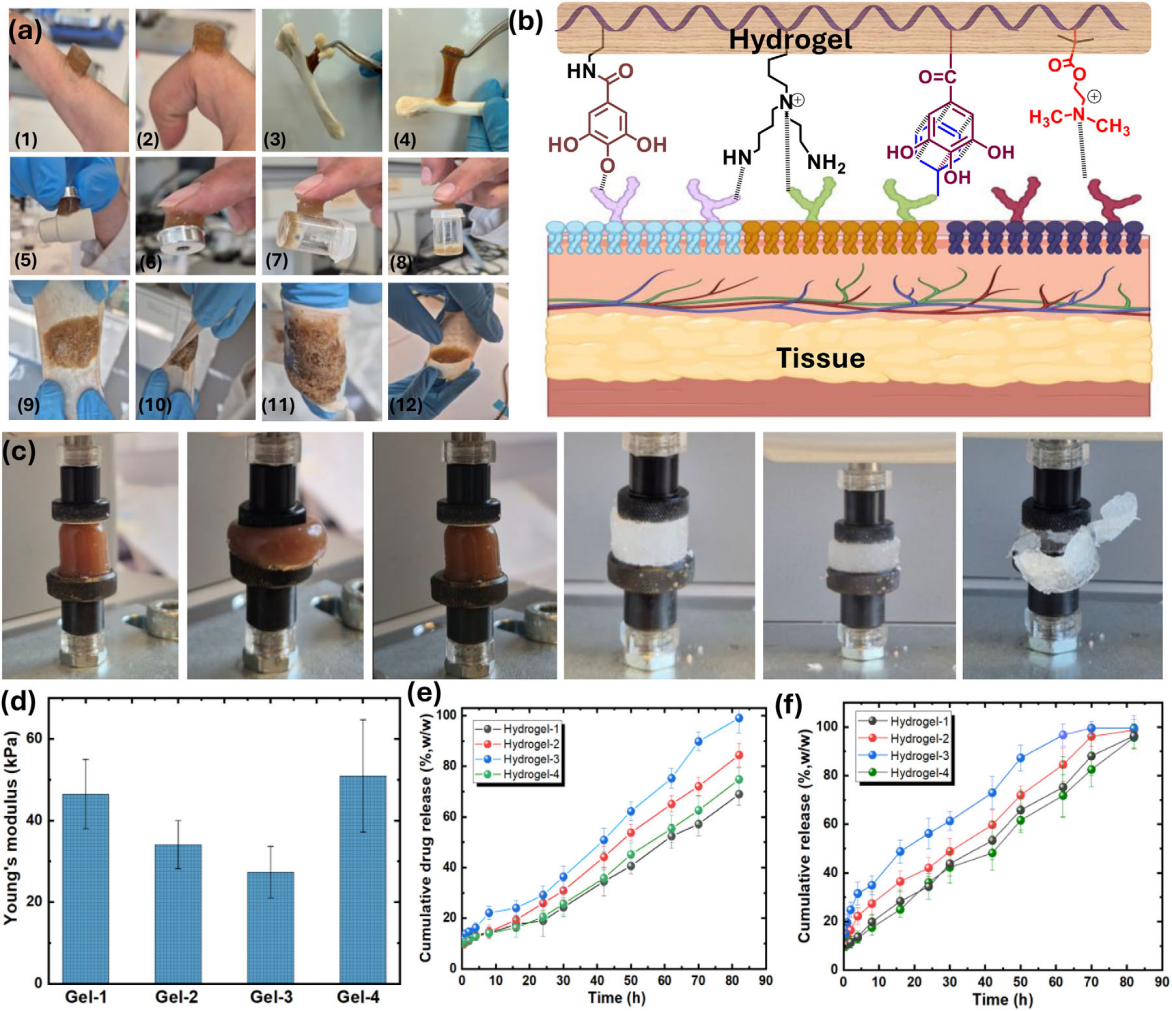
Hydrogel Adhesion: (a‐1‐2) Demonstrating hydrogel adhesion to human skin under static and motion conditions. (a‐3‐4) Showcasing adhesion between bone and bone, and bone and hydrogel under tensile conditions. (a‐5‐8) Adhesion to metal‐rubber and metal‐skin, metal‐glass, and metal‐plastic surfaces. (a‐9‐12) Hydrogel adhesion with chicken skin under various twisting conditions. (b)Schematic illustration of the Cl‐Plu‐Cl/PDMA/PEI‐GA hydrogel showing cationic PDMA and PEI chains engaging in ionic and π–cation interactions with tissue phospholipids and proteins, together with GA‐mediated hydrogen bonding at the tissue–hydrogel interface. (c) hydrogels Elastic Properties Demonstration of hydrogel's elastic and brittle properties: (upper panel) Gel‐4 exhibits brittle behavior, while (lower panel) Gel‐1 shows elastic properties. (d) Graph showing the Young's modulus of the hydrogels, highlighting the varying elastic properties of the different gel formulations. (e,f) cumulative release of Amoxicillin from the hydrogel at pH 7.4 and 5, respectively. The release medium was aqueous PBS in all the experiments.

To assess the bioadhesive performance of our injectable hemostatic hydrogels, we conducted adhesion tests under static and dynamic conditions. As illustrated in Figure 6(a‐1), (a‐2), the hydrogels adhered robustly to human skin, even under static contact and dynamic motions, such as finger extension and contraction. Figure 6a Panels (a‐3 to a‐4) demonstrate adequate tensile adhesion between bone‐bone and bone‐hydrogel interfaces. In contrast, Figure 6, panel (a‐5 to a‐8), shows strong adhesion across diverse substrates, including metal‐rubber, metal‐skin, metal‐glass, and metal‐plastic interfaces. Moreover, the hydrogels maintained firm adhesion to chicken skin under twisting forces (Figure 6, panels a‐9 to a‐12). Under physiologically relevant wet conditions, after applying the hydrogels to PBS‐hydrated chicken skin (37°C, 30 min) and incubating for an additional 30 min, the hydrogels resisted gentle agitation and remained adhered. This confirms strong interfacial adhesion in moist environments, similar to those found in vivo. Further testing on other relevant surfaces‐polypropylene, glass, stainless steel, rubber, wood, and bone‐showed stable adhesion under wet conditions (Figure 6a), indicating the hydrogels’ adaptability and binding capacity across a range of materials commonly found in clinical contexts. The hydrogels thus demonstrate robust, effective sealing performance even under challenging physiological conditions. The Cl‐Plu‐Cl/PDMA/PEI‐GA hydrogel demonstrated strong wet tissue adhesion, shown by high lap shear strength and solid peel resistance on porcine sausage skin, as well as burst pressures surpassing physiological arterial levels, indicating stable sealing under dynamic conditions. As shown in Figure 6a, this performance results from the combination of cationic PDMA and PEI segments forming ionic and π–cation interactions with negatively charged cell membranes and tissue components, along with GA‐mediated hydrogen bonding and hydrophobic interactions at the tissue–hydrogel interface. The synergy between these interfacial interactions and the highly crosslinked, cohesive hydrogel network enables close tissue contact and effective load transfer, turning the molecular adhesion into macroscopic mechanical strength and hemostatic effectiveness. These findings collectively show that the engineered hydrogel design provides strong and durable adhesion suitable for hemostatic sealing in complex wound environments. The Cl‐Plu‐Cl/PDMA/PEI‐GA hydrogel showed excellent wet‐tissue adhesion, evidenced by high lap‐shear strength and firm peel resistance on sausage casing substrates, along with burst pressures above normal arterial levels, indicating reliable sealing under dynamic conditions. As demonstrated in Figure 6a, this performance arises from the combination of cationic PDMA and PEI segments forming ionic and π–cation interactions with negatively charged cell membranes and tissue components, along with GA‐mediated hydrogen bonding and hydrophobic interactions at the tissue–hydrogel interface. The synergy of these interfacial interactions and the highly crosslinked, cohesive hydrogel network promotes close tissue contact and effective load transfer, transforming molecular adhesion mechanisms into macro‐level mechanical strength and hemostatic function.

### 3.9 Hydrogel Bulk Mechanical Properties

To investigate the influence of the Cl‐Plu‐Cl/PDMA and PEI(GA) ratio on the bulk mechanical properties and adhesion strength of the hydrogels, we conducted mechanical tests, including compression and 90° peel tests. According to (Figure 6b; Movies S5 and S6), the compression test tensile stress‐strain curves revealed that Gel‐1 to Gel‐3 showed better compressibility, stress resistance, and durability in cyclic properties. However, Gel‐4 did not show results similar to those of Gel‐1. The increased crosslinking density reduces compressibility by restricting chain mobility, thereby enhancing rigidity and increasing the gel modulus. This collective effect decreases both compressibility and elasticity, resulting in lower cyclic compressibility [56].

The mechanical stiffness of the hydrogels, quantified by Young's modulus, showed significant variation across formulations. Gel‐1 and Gel‐4 exhibited higher moduli of 46.5 kPa (±8.5) and 50.94 kPa (±13.75), respectively, whereas Gel‐2 and Gel‐3 showed lower values of 34.11 kPa (±5.9) and 27.35 kPa (±6.33) (Figure 6c). This variation reflects differences in crosslinking density and internal network architecture, which directly influence the mechanical properties and porosity of hydrogels [57, 58]. Higher Young's modulus values correspond to more densely crosslinked polymer networks, consistent with SEM observations of continuous and compact polymer walls in Gel‐1 and Gel‐4. In contrast, the lower stiffness in Gel‐2 and Gel‐3 aligns with their more porous, fibrous microstructure, indicative of reduced crosslinking [59]. These results demonstrate that tuning the crosslinking chemistry effectively modulates hydrogel stiffness, a critical factor for optimizing hydrogels for biomedical applications such as tissue engineering and controlled drug delivery [60].

The increase in hydrogel stiffness with increasing amounts of Cl‐Plu‐Cl and PEI(GA) crosslinkers is primarily due to the higher crosslink density, which improves mechanical strength, flexibility, and compressive recoverability [60, 61]. However, excessive crosslinking severely restricts the mobility of hydrophobic segments within the polymer network, resulting in brittle hydrogels that are prone to rupture during mechanical stress (Figure 6b) [62]. In contrast, conjugation of PEI with gallic acid (GA) introduces dynamic hydrogen bonding and electrostatic interactions through phenolic groups, creating a more flexible and adaptable network [57]. These dynamic interactions, particularly *π*–*π* and π–cationic bonds, play a crucial role in reducing brittleness and enhancing the elasticity of the hydrogels [63, 64]. Such flexibility is critical for hemostatic injectable hydrogels, as it facilitates tissue conformance, dynamic mechanical adaptability, and clot stability, ensuring effective hemostasis and seamless integration with wound sites.

Among the tested formulations, Gel‐1, Gel‐2, and Gel‐3 exhibited elastic behavior, with Gel‐3 showing the highest elasticity due to its relatively lower crosslinking density. This reduced crosslinking enables greater polymer chain mobility, resulting in improved deformation and recovery under load. Gel‐3's Young's modulus was the lowest at 27.35 ± 6.33 kPa, consistent with its superior elastic nature. On the other hand, Gel‐4, despite exhibiting the highest stiffness with a Young's modulus of 50.94 ± 13.75 kPa, behaved in a brittle manner due to excessive crosslinking that limited chain movement and compromised recoverability. Gel‐1 and Gel‐2 showed intermediate stiffness values (46.5 ± 8.5 kPa and 34.11 ± 5.9 kPa, respectively), corresponding to their moderate elasticity (Figure 6c). These results highlight the delicate balance between crosslinking density and elasticity necessary to optimize hydrogel mechanical properties.

Further compressive testing demonstrated that Gel‐4 had the highest compressive strength, consistent with its increased stiffness (Figure 6c). However, cyclic compression tests revealed that Gel‐1 could repeatedly recover its shape, indicating excellent resilience. At the same time, Gel‐4 fractured under similar loading conditions, underscoring its brittleness. Gel‐2 and Gel‐3 exhibited higher compressive stress and strain than Gel‐1, reflecting improved mechanical performance through balanced crosslinking and flexibility (Figure 6b). The superior compressive properties of Gel‐4 are attributed to the reinforcing effect of PEI(GA), which simultaneously increases stiffness and provides some flexibility to the network. Overall, these findings demonstrate that incorporating PEI(GA) enables fine‐tuning of hydrogel mechanical properties by balancing stiffness and elasticity. This balance is crucial for injectable hydrogels to maintain their structural integrity while providing the flexibility necessary for tissue integration and dynamic physiological environments.

### 3.10 In Vitro Drug Loading and Release Behavior

Amoxicillin, a hydrophilic *β*‐lactam antibiotic, was incorporated into Cl‐Plu‐Cl/PDMA/PEI(GA) hydrogels during sol‐gel transition without disturbing gelation kinetics or mechanical properties. FT‐IR and UV–vis spectroscopy (Figure S10a,b) confirmed the structural integrity of the drug after loading, underscoring the robust physicochemical compatibility with the polymeric matrix. Loading efficiency reached 94.6 ± 2.1% (*n* = 3), signifying efficient entrapment and negligible leakage.

In vitro release assays unveiled pH‐responsive, sustained profiles (Figure 6d,e). At pH 7.4, cumulative release spanned 69%–99% over 80 h (mean 81%), whereas at pH 5 (emulating inflamed tissues), it surged to 96%–100% (mean 98%), driven by PEI(GA) protonation‐induced swelling and pore expansion. Profiles conformed to zero‐order kinetics at pH 7.4 (R^2^ = 0.995–0.999; Table S2), exhibiting concentration‐independent elution conducive to steady‐state therapeutics. At pH 5, zero‐order linearity persisted (R^2^ = 0.913–0.996); however, the Higuchi (R^2^ > 0.96) and Korsmeyer–Peppas (R^2^ > 0.93) models more accurately delineated the diffusion–erosion interplay. Zero‐order rates ranked Gel‐3> Gel‐2> Gel‐4> Gel‐1 at pH 7.4 and Gel‐2 ≈ Gel‐4> Gel‐3> Gel‐1 at pH 5, corresponding swelling ratios (Figure 5a) and degradation patterns (Figure 5b). By 80 h at pH 7.4, Gel‐1 and Gel‐3 released 69% and 99% of entrapped amoxicillin, respectively. Korsmeyer–Peppas exponents (*n* = 0.32–0.41) affirmed Fickian diffusion predominance. Moderate first‐order (R^2^ > 0.78) and Hixson–Crowell (R^2^ > 0.92) correlations, with accentuated negative slopes at pH 5, suggest that matrix erosion is augmented. The accelerated acidic efflux of the hydrophilic payload aligns with the amplified solubilization in swollen hydrophilic domains. In aggregate, these attributes, superior loading, pH‐gated kinetics, and extended elution, render the hydrogels compelling for targeted, infection‐adaptive antibiotic delivery [65, 66]. The distinct release profiles among Gel‐1–Gel‐4 arise from differences in their Cl‐Plu‐Cl/PDMA/PEI(GA) ratios, which modulate crosslinking density, network microstructure, and degradation: more densely crosslinked, less swollen formulations (e.g., Gel‐1 and Gel‐4) present smaller mesh sizes and slower, more sustained diffusion, whereas more loosely crosslinked gels with higher swelling and faster degradation (e.g., Gel‐2 and Gel‐3) permit faster Fickian diffusion and erosion‐assisted release, consistent with the observed ranking of zero‐order rates at both pH 7.4 and pH 5. The faster and more complete release at mildly acidic pH arises from enhanced protonation of PDMA tertiary amines and PEI units, which increases electrostatic repulsion within the network, swelling, and mesh size, thereby facilitating diffusion of the encapsulated amoxicillin, whereas at physiological pH the lower degree of protonation maintains a more compact network structure with reduced mesh size, leading to slower, sustained diffusion‐controlled release that is further modulated by the formulation‐dependent crosslinking density of each gel. This protonation‐regulated transition from a relatively collapsed state at pH 7.4 to a highly swollen, partially relaxed state at pH 5.0, where increased chain mobility and water uptake enhance both Fickian diffusion and erosion‐assisted transport of hydrophilic drugs, is consistent with pH‐responsive release mechanisms described for amine‐containing nanocomposite [67]. The quicker and more complete release at mildly acidic pH results from increased protonation of PDMA tertiary amines, which boosts electrostatic repulsion within the network, leading to swelling and larger mesh size, and thus enables easier diffusion of the encapsulated amoxicillin.

### 3.11 In Vitro Antibacterial Activity of Amoxicillinloaded‐Hydrogels

The antimicrobial performance of amoxicillin‐loaded Cl‐Plu‐Cl/PDMA/PEI(GA) hydrogels was evaluated against *S. aureus* and MRSA using agar diffusion and suspension‐killing assays (Figure S11). Without drug loading, the hydrogels showed no visible inhibition zones on either bacterial lawn, despite their cationic nature, while amoxicillin‐loaded gels produced distinct, clear zones, confirming the release of active antibiotic from the matrix (Figure S11a)). For *S. aureus*, the inhibition zone was approximately 32 mm, whereas a smaller zone of approximately 10 mm appeared against MRSA, consistent with MRSA's lower inherent susceptibility to amoxicillin. CFU analysis revealed that drug‐free hydrogels caused only a minor decrease in viable bacteria compared to the control. In contrast, amoxicillin‐loaded gels decreased *S. aureus* viability by approximately 99.99% and MRSA viability by 82.72% (Figure S11b,c). These findings show that the hydrogel system can efficiently deliver amoxicillin in vitro and significantly inhibit the growth of crucial Gram‐positive wound pathogens, supporting its potential as an antimicrobial hemostatic dressing and as a flexible platform for loading other therapeutic agents.

### 3.12 Injectable Hemostatic Hydrogels Show Strong Tissue Adhesion and Burst Pressure Properties

#### 3.12.1 Bioadhesive Performance and Burst Pressure of Hydrogels

**FIGURE 7:**
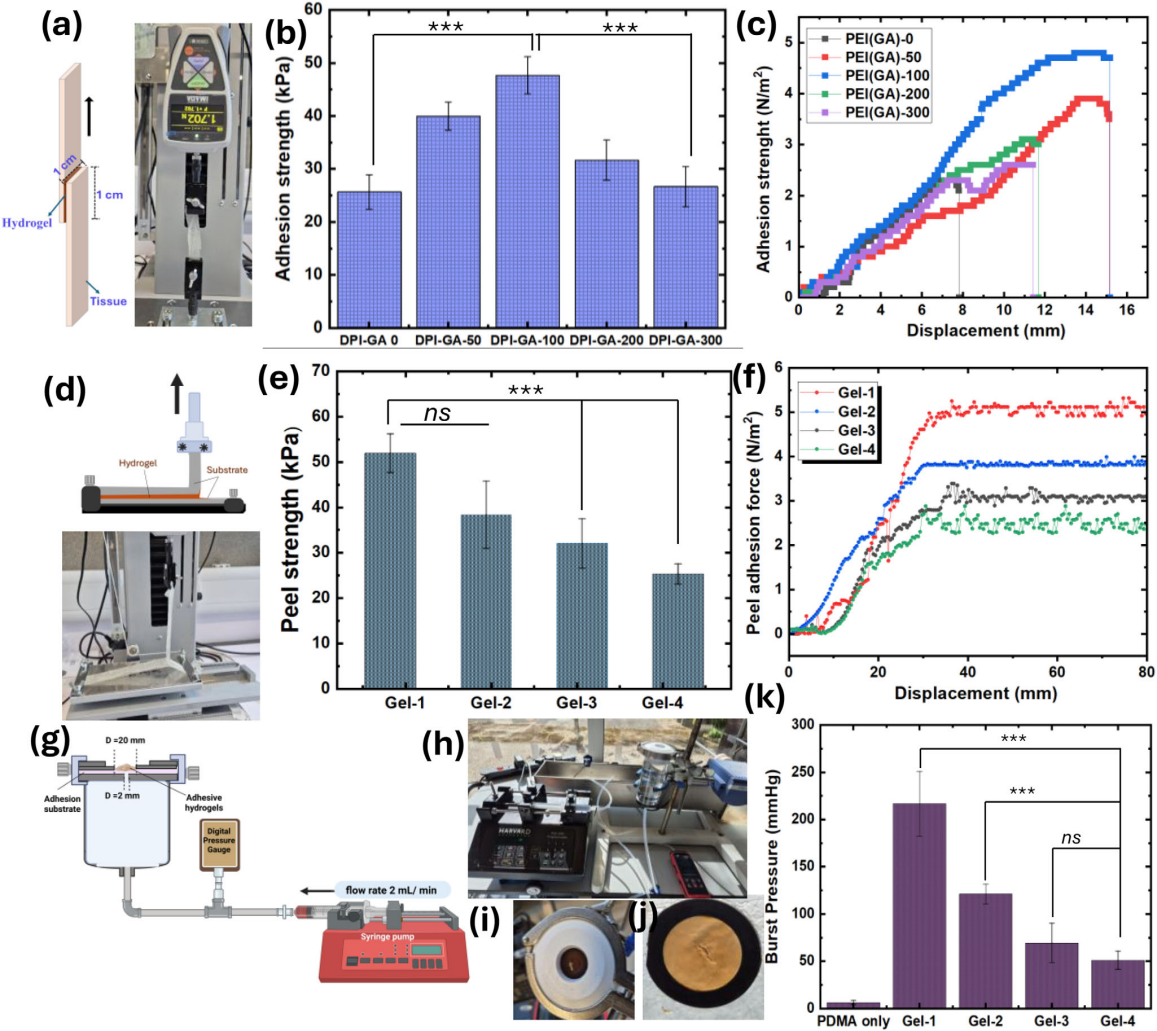
Evaluation of bioadhesion and burst pressure resistance of PDMA/PEI(GA)/Cl‐Plu‐Cl hydrogels using porcine sausage skin as a tissue model. (a) Illustration of the lap‐shear adhesion test setup. (b) Adhesion strength of Gel‐1 with varying concentrations of PEI(GA), showing the influence of crosslinker content. (c) Corresponding force–displacement curves obtained from lap‐shear tests. (d) Schematic diagram and image of the 180° peel test setup. (e) Peel strength comparison of Gel‐1 to Gel‐4 hydrogels on porcine skin. (f) Peel force–displacement curves for each hydrogel formulation. (g) Schematic of the burst pressure test setup using a syringe pump and pressure gauge. (h) Photograph of the experimental setup used for burst pressure testing. (i,j) Representative image of a hydrogel applied over a pinhole on porcine sausage skin. (k) Comparison of burst pressure across PDMA‐only and Gel‐1 to Gel‐4 hydrogels. (*n* = 4) Data are presented as mean ± standard deviation. Statistical analysis performed using one‐way ANOVA: ^*^ *p* < 0.05, ^**^ *p* < 0.01, ^***^ *p* < 0.001.

The lap shear adhesion strength of Cl‐Plu‐Cl/PDMA/PEI(GA) injectable hydrogels was evaluated on porcine sausage skin following a modified ASTM F2255‐05 protocol under fully hydrated conditions (PBS, 37°C, one h curing). Optimization of PEI(GA) content revealed a clear dependence of adhesion strength on crosslinker concentration. Hydrogels without PEI(GA) exhibited a lap shear strength of 25.7 ± 3.2 kPa, whereas increasing PEI(GA) to 50 and 100 mg mL^−^^1^ significantly enhanced adhesion to 40.0 ± 2.6 kPa and 47.7 ± 3.5 kPa, respectively (*p* < 0.01 vs. PEI(GA)‐free). Beyond this optimum, adhesion strength decreased to 31.7 ± 3.8 kPa at 200 mg mL^−^^1^ and 26.7 ± 3.8 kPa at 300 mg mL^−^^1^, (Figure 7a) likely due to excessive gallic acid‐mediated aggregation causing reduced surface wettability and impaired interpenetration with tissue fibers [68, 69]. The strong interfacial bonding at the optimal PEI(GA) concentration is attributed to synergistic covalent imine linkages and non‐covalent interactions between the hydrogel network and tissue collagen.

The peeling adhesion strength of the injectable hydrogels, measured on porcine sausage skin under hydrated conditions, showed apparent differences between formulations (Figure 7c,d). Gel‐3 exhibited the highest peeling force (51.96 ± 1.49 kPa), followed by Gel‐2 (38.40 ± 1.39 kPa), Gel‐1 (32.08 ± 1.41 kPa), and Gel‐4 (25.31 ± 2.25 kPa) (Figure 7b). The superior performance of Gel‐3 can be attributed to its optimal PEI(GA) content, which promotes a balanced density of covalent imine bonds and non‐covalent interactions with tissue collagen. This allows for both strong anchoring and sufficient flexibility to dissipate peeling stresses without premature failure. In contrast, Gel‐4, which lacks PEI(GA), relied primarily on physical hydrogen bonding and hydrophobic interactions, resulting in weaker interfacial anchoring and lower peeling energy. Interestingly, Gel‐1 and Gel‐2 also demonstrated lower adhesion than Gel‐3 despite containing PEI(GA), likely due to sub‐optimal crosslinking densities. This aligns with previous reports that excessive or insufficient aldehyde‐mediated crosslinking can compromise interfacial bonding by either reducing tissue wetting ability or limiting polymer chain interpenetration into the substrate [68, 69]. The force‐extension curves (Figure 7c) further supported these findings: Gel‐3 displayed smooth, continuous force profiles without abrupt drops, indicative of cohesive gel failure rather than interfacial delamination. Such homogeneous interface formation is advantageous for biomedical sealants, as it minimizes the risk of leakage under dynamic tissue deformation [70, 71].

The burst pressure is a critical parameter in evaluating the performance of hemostatic materials, as it reflects the material's ability to withstand the hydrostatic pressure exerted by blood at a wound site. Effective hemostatic sealants must resist pressures exceeding normal and elevated physiological blood pressures to prevent re‐bleeding and ensure wound closure. This requires a delicate balance between the material's bulk mechanical strength (cohesion) and its adhesive interaction with tissue surfaces. Both properties work synergistically: strong adhesion prevents sealant detachment, while robust cohesion maintains structural integrity under stress. In this study, burst pressure testing was performed according to ASTM F2392‐04 using porcine sausage skin with a standardized pinhole defect to simulate vascular injury, as illustrated in (Figure 7g–j). The PDMA‐only hydrogel exhibited minimal burst pressure (6.25 ± 2.22 mmHg), insufficient for effective hemostasis. In contrast, the injectable hydrogels incorporating PEI(GA) showed substantially improved performance (Figure 7k). Gel‐1 demonstrated the highest burst pressure (216.75 ± 34.27 mmHg), followed by Gel‐2 (161.25 ± 10.44 mmHg), Gel‐3 (89.5 ± 20.86 mmHg), and Gel‐4 (69.5 ± 20.86 mmHg), all exceeding normal human systolic pressure (∼120 mmHg).

Analysis of failure modes during burst testing revealed cohesive failure in Gel‐1, Gel‐2, and Gel‐3, in which the hydrogel bulk ruptured while adhesion to tissue remained intact. This indicates strong interfacial bonding coupled with a mechanical limit within the gel network. In contrast, Gel‐4 exhibited adhesive failure, detaching from tissue due to insufficient adhesion, likely caused by the absence or reduced content of PEI(GA). These results emphasize that optimal hemostatic hydrogels must strike a balance between crosslinking density and polymer composition to maximize both adhesion strength and network elasticity. Such synergy enables the hydrogel to withstand physiological and traumatic pressures without detaching or rupturing, which is crucial for reliable wound sealing and bleeding control. Furthermore, the burst pressures achieved here notably surpass those of conventional fibrin‐based sealants (typically 30–160 mmHg) [35, 36, 72] positioning these hydrogels as strong candidates for advanced hemostatic applications where high‐pressure arterial sealing is required.

#### 3.12.2 Effect of pH on Mechanical Strength and Adhesion

To further evaluate how pH modulates the bulk and interfacial properties of the hydrogels, samples were formed at pH 7.4 and subsequently equilibrated either at pH 7.4 or pH 5.0 before testing. Hydrogels incubated at pH 5.0 showed a modest decrease in compressive modulus and failure stress but a slight increase in lap‐shear adhesion on wet tissue compared with pH 7.4, consistent with increased swelling yet stronger electrostatic interactions with negatively charged tissue surfaces (Figure S12). These observations support that mild acidic environments can soften the hydrogel while simultaneously enhancing tissue adhesion, which is beneficial for hemostasis at inflamed wound sites [73].

#### 3.12.3 Excellent In Vitro Blood Clotting of the Injectable Hydrogels

**FIGURE 8:**
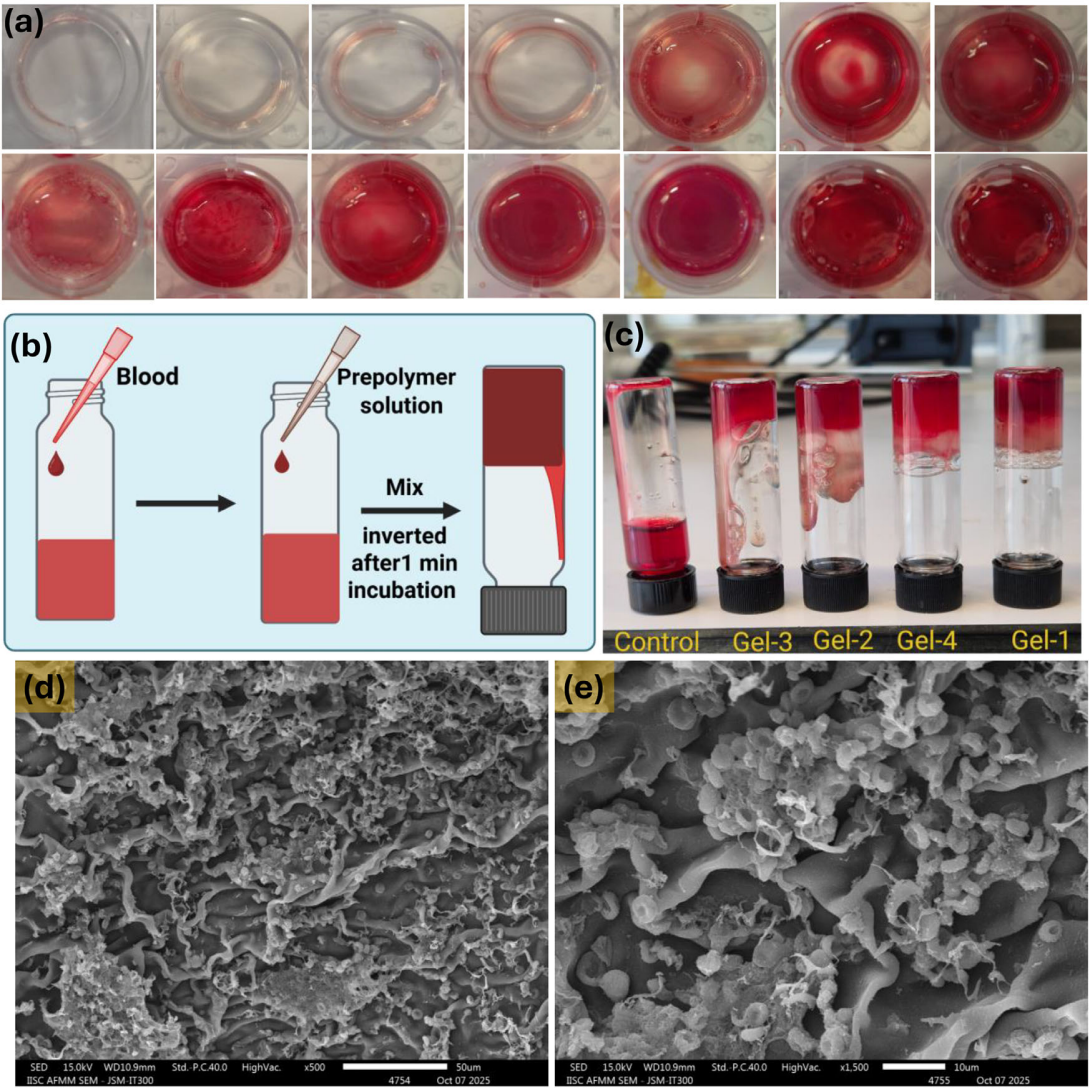
In vitro hemostatic performance of hydrogels. (a) Photographs showing coagulation of sheep blood added to the uncoated (control) wells or hydrogel‐coated wells at different time points. (b) Schematic of the in vitro hemostasis assay, where blood and prepolymer solution are mixed and subjected to inversion testing after one minute of incubation. (c) Photographic images of in vitro clot formation in control and different gel formulations (Gel‐1 to Gel‐4), with Gel‐1 to Gel‐4 showing robust clot retention compared to the control. (d,e) Scanning electron micrograph of the lyophilized Gel‐1 hydrogel used for hemostasis. The image shows pronounced adhesion and aggregation of red blood cells (RBCs) on the porous microstructure, confirming strong blood‐material interaction and effective hemostatic activity.

Effective hemostatic materials should rapidly promote blood coagulation while minimizing damage to blood cells. In the presence of the Cl‐Plu‐Cl/PDMA/PEI(GA) hydrogels, heparinized whole sheep blood formed a stable hydrogel–blood composite within about 1 min that remained firmly attached to the vial bottom upon inversion, whereas the blood–fibrin gel mixture showed noticeable downward flow of uncoagulated blood, indicating less efficient clot stabilization under the same conditions (Figure 8a). Gel‐1 and Gel‐4 retained the blood clot most effectively with negligible spillage, while Gel‐2 and Gel‐3 showed slight leakage yet still demonstrated rapid clot formation, highlighting the strong overall coagulation capacity of all formulations.​Clotting time measurements further confirmed the superior hemostatic performance of the injectable hydrogels. In uncoated control wells, sheep blood coagulated after approximately 8–9 min. In contrast, all hydrogel‐coated wells significantly shortened the clotting time, with no statistically significant differences among Gel‐1, Gel‐2, Gel‐3, and Gel‐4 (Figure 8b). These results indicate that the Cl‐Plu‐Cl/PDMA/PEI(GA) hydrogels not only induce rapid clot formation but also maintain stable clot adherence, supporting their potential for urgent hemostatic applications in surgical and trauma settings.

#### 3.12.4 Excellent in Vitro and in Vivo Hemostasis Properties of the Hydrogels

Effective hemostatic materials must facilitate rapid and efficient blood clotting to staunch hemorrhage. To assess the hemostatic efficacy of our injectable hydrogels, we evaluated their blood coagulation kinetics using heparinized sheep whole blood. Clotting times were quantified following addition to hydrogel‐coated 24‐well plates. Uncoated control wells required ∼7–8 min for coagulation (Figure 8a). By comparison, clot formation was substantially hastened in wells coated with injectable hydrogels or fibrin gels (Figure 8a). However, the direct admixture of the Cl‐Plu‐Cl/PDMA/PEI(GA) prepolymer solution with blood in vials prompted rapid in situ gelation, producing a stable clot that adhered tenaciously to the vial base upon inversion. In the control, however, pronounced blood effusion occurred upon inversion, denoting deficient clot coherence (Figure 8b,c). The clotting time in injectable hydrogel‐coated wells (180 s) closely mirrored that in fibrin gel‐coated wells (200 s) in prior reports [40], showing no statistically significant divergence. Prepolymer blood mixtures underwent inversion testing after a 1‐min incubation (Figure 8a,b). All hydrogel variants (Gel‐1 to Gel‐4) yielded resilient, inversion‐resistant clots that far surpassed the control (Figure 8c). These observations reveal how the hydrogels' cationic moieties and dual crosslinking enhance clotting proficiency by promoting platelet activation and RBC aggregation, as shows in SEM images of blood coagulated Hydrogel‐1 (Figure 8d,e), thus supporting their potential for in vivo hemostatic deployment.

**FIGURE 9:**
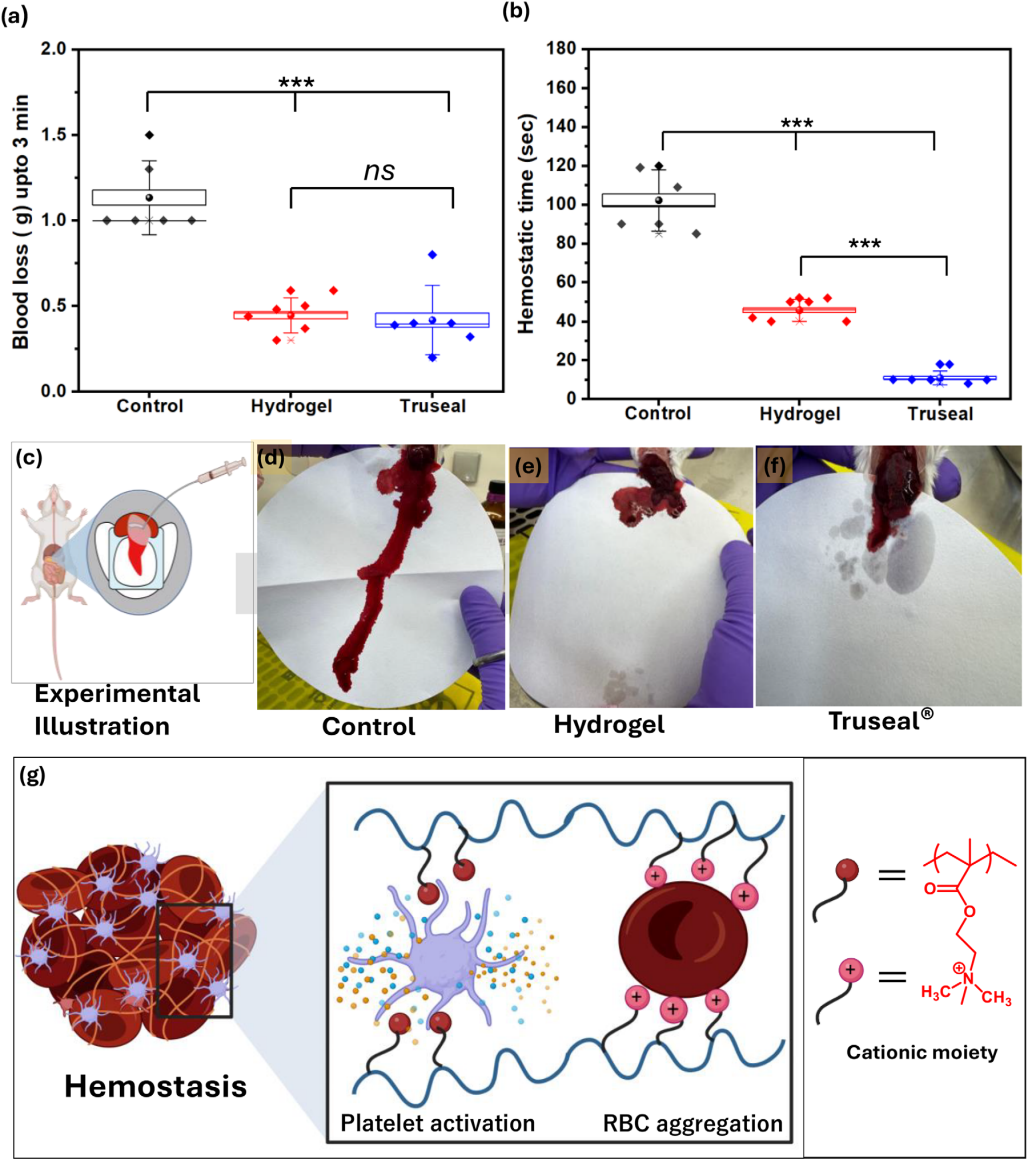
In vivo hemostatic performance of hydrogels. (a) Quantification of blood loss (g, up to 3 min) in an in vivo mice liver hemorrhage model, showing significant reduction with hydrogel and Truseal compared to control (^***^ *p* < 0.001, n.s. = not significant). (b) Hemostatic time (sec) in the in vivo liver injury model, demonstrating markedly faster clotting with hydrogel and Truseal treatment compared to control (^***^ *p* < 0.001). (c) Schematic illustration of the in vivo mice liver hemorrhage model. (d–f) Representative images of bleeding outcomes in control, hydrogel, and Truseal‐treated groups, respectively, in the in vivo model. (g) Proposed mechanism of Truseal‐mediated hemostasis, involving platelet activation and red blood cell (RBC) aggregation facilitated by cationic functional groups. (*n* = 6) per group; statistical analysis by one‐way ANOVA with Tukey's post‐hoc test).

We next examined the in vivo hemostatic efficacy of our injectable hydrogels using a representative formulation (Hydrogel‐1) and comparing it to the commercially available hemostatic adhesive Truseal and a control (no material) group. Hemostatic times were observed and manually recorded for the hydrogels, Truseal, and the control. The hemostatic efficacy of the Cl‐Plu‐Cl/PDMA/PEI(GA) hydrogels was assessed by applying the gel directly to the bleeding site on a resected liver and quantifying blood loss after hemostasis. As shown in (Figure 9a,b), the hydrogel reduced blood loss by 61% ± 8% and 63% ± 17% compared to the control (no materials) group (*p* < 0.05, *n* = 6), demonstrating comparable efficacy with greater consistency to commercial materials. To demonstrate the hydrogels' ability to sustain against the high hydrostatic pressure of blood flow from blood vessels, we established a mouse liver hemorrhage model, as illustrated in Figure 9c. Remarkably, the hydrogel's performance matches that of Truseal, Fibrin glue, a clinically established sealant hemostat, despite lacking clotting factors like thrombin, as shown in (Figure 9d,e,f) [35, 74, 75]. This suggests that the hydrogel facilitates rapid hemostasis primarily through robust tissue adhesion and physical sealing, consistent with reports on adhesive hydrogels that prioritize mechanical barriers over biochemical coagulation [35, 74].

To elucidate the hemostatic mechanism, we tested individual components (20 wt/v% Cl‐Plu‐Cl or 20 wt/v% PDMA/PEI(GA)) in the same model. Neither component alone exhibited significant hemostatic activity, emphasizing the synergistic role of Cl‐Plu‐Cl, PDMA, and gallic acid‐functionalized polyethyleneimine (PEI(GA)), which generates a permanent cationic charge through a crosslinking reaction. Strong tissue adhesion underpins the hydrogel's efficacy and arises from the cationic charge of chloride‐terminated Pluronic F‐127, the tertiary amine groups of PDMA, and the bioadhesive functionality of gallic acid. These cationic moieties enable electrostatic interactions with negatively charged tissue surfaces, forming a durable physical barrier that seals bleeding sites. Additionally, PDMA promotes platelet activation and aggregation, a critical step in the coagulation process, by interacting with anionic platelet surfaces, as observed in similar cationic formulations (Figure 9g) [71, 76, 77]. This dual mechanism, combining adhesive sealing and biochemical platelet activation, enhances the hydrogel's hemostatic performance. The hydrogel's dual crosslinking mechanism further amplifies its hemostatic and wound‐healing potential. Chemical crosslinking via nucleophilic substitution between PDMA and Cl‐Plu‐Cl, together with physical crosslinking driven by the thermoresponsive behavior of Pluronic F‐127, enables the hydrogels to form rapidly in situ (1–4 min) and achieve a high gel fraction (96–99%) under physiological conditions (pH 7.4, 37°C), while generating only minimal heat (∼1°C per gram) [47]. This imparts mechanical stability, shear‐thinning behavior, and enhanced platelet adhesion, akin to dually crosslinked PEG/PDMA‐*b*‐PNIPAM hydrogels [47].

These properties make the hydrogel ideal for dynamic wound environments requiring rapid gelation and robust adhesion. In vivo hemostatic assays corroborated the in vitro findings. To evaluate the clinical translatability of the hydrogel, we tested its delivery via single‐barreled syringe systems. Single‐syringe application significantly reduced bleeding compared to the control (*p* < 0.05), matching Truseal delivered via a single‐barrelled syringe (Figure 9C). Unlike covalently crosslinked hydrogels, which require separate prepolymers solutions [78], the Cl‐Plu‐Cl/PDMA/PEI(GA) hydrogel's dual crosslinking allows premixing and single‐syringe delivery, simplifying application and enhancing surgeon operability. This mirrors physically crosslinked systems like nanocomposite gels [79], yield stress fluids [48], and self‐assembling peptide nanofibrous hydrogels [80], which enables single‐solution injection. The hydrogel's shear‐thinning and self‐healing properties facilitate easy injection and conformal adaptation to irregular tissue surfaces [47, 81]. Robust wet adhesion, driven by cationic interactions and gallic acid's bioinspired adhesive properties, enables the hydrogel to effectively control internal bleeding in moist environments. This strong adhesion supports its use in endoscopic and laparoscopic procedures for minimally invasive hemorrhage management.

Compared to previously reported injectable hydrogels, our Cl‐Plu‐Cl/PDMA/PEI(GA) hydrogel system uniquely combines rapid, initiator‐free gelation, strong wet‐tissue adhesion, high burst pressure, and pH‐responsive drug delivery within a single platform. These chemistry‐driven design features set our hydrogel apart from many existing injectable sealants that rely on external initiators or offer either strong adhesion or controlled release, but not all of these functions in a single degradable platform. For additional context, Table S1, summarizes the compositions, advantages, disadvantages, and reported adhesion strengths and burst pressures of representative commercial fibrin, PEG/protein, and topical hemostatic products alongside the present hydrogel, further emphasizing the combined mechanical and hemostatic benefits of the Cl‐Plu‐Cl/PDMA/PEI‐GA system. The prepolymer solution forms a cohesive network within 1–4 min at 37°C and physiological pH without external initiators, while remaining readily injectable through long 21–23 G tubing at clinically feasible forces (2–4 N), which is advantageous for laparoscopic or catheter‐based delivery. Under fully hydrated conditions, the optimized formulation achieves lap‐shear strengths up to about 48 kPa and peel strengths around 52 kPa on porcine skin, and withstands burst pressures of approximately 220 mmHg, substantially higher than typical fibrin sealants (≈30–160 mmHg) and above normal arterial pressure, indicating reliable sealing in high‐pressure bleeding scenarios. In parallel, the network provides high amoxicillin loading efficiency (∼95%) and pH‐gated release, with 69%–99% cumulative release over ∼80 h at pH 7.4, compared with ∼98% at mildly acidic pH, enabling microenvironment‐responsive therapy at inflamed wound sites. Finally, incorporation of gallic‐acid‐functionalized PEI confers potent antioxidant activity in DPPH assays, >90% fibroblast viability, low hemolysis, and complete in vitro degradation within 3–4 weeks, while in vivo the hydrogel reduces blood loss by ∼61% and achieves hemostatic efficacy comparable to Truseal despite lacking exogenous clotting factors.

Collectively, this combination of minimally invasive delivery, dual crosslinking, robust wet adhesion and burst resistance, pH‐responsive drug release, antioxidant functionality, and controlled degradability distinguishes the present system from existing injectable hydrogels and highlights its translational potential for trauma and surgical care. Overall, the combination of nucleophilic crosslinking between PDMA and Cl‐Plu‐Cl, GA‐mediated multi‐modal adhesion, and pH‐responsive cationic chemistry explains the integrated performance of this system, namely rapid in situ gelation, strong wet adhesion, burst resistance, and microenvironment‐dependent drug release. These chemistry‐driven design features distinguish the present Cl‐Plu‐Cl/PDMA/PEI(GA) hydrogel from many existing injectable sealants that rely on external initiators or provide either strong adhesion or controlled release, but not all of these functions within a single degradable platform.

In summary, the Cl‐Plu‐Cl/PDMA/PEI(GA) hydrogel exhibits exceptional hemostatic performance and burst pressure comparable to fibrin glue and Truseal through strong tissue adhesion, cationic charge‐mediated platelet activation, and dual crosslinking. Its compatibility with single‐syringe and minimally invasive delivery enhances its versatility for clinical applications, from trauma surgery to internal hemorrhage control. These properties, backed by robust in vitro and in vivo data, position the hydrogel as a promising candidate for advanced wound management.

## 4 Conclusion

In this study, we developed a novel injectable, pH‐responsive amphiphilic hydrogel, Cl‐Plu‐Cl/PDMA/PEI(GA). The hydrogel forms through an initiator‐ free nucleophilic substitution between PDMA, chloride‐ terminated Pluronic F‐ 127, and gallic acid‐ functionalized branched polyethyleneimine. It undergoes rapid in situ gelation (∼54 s) across physiologically relevant pH values (5–7.4). The resulting shear‐thinning, viscoelastic network is suitable for minimally invasive delivery into irregular, dynamically bleeding traumatic wounds. Systematic in vitro and in vivo evaluation, including a mouse liver hemorrhage model, demonstrated the hydrogel's strong hemostatic performance. It reduced blood loss by approximately 61% (compared to about 63% for Truseal), provided strong wet‐ tissue adhesion (∼47 kPa lap‐shear strength), and resisted burst pressures up to approximately 220 mmHg‐ exceeding normal arterial pressure and ensuring reliable sealing under flow. The pH‐responsive design enabled controlled antimicrobial release, with about 60% of the cumulative amoxicillin released over 80 h at pH 7.4. The hydrogel exhibited robust in vitro antibacterial activity against *S. aureus* and MRSA, as confirmed by CFU reduction and live/dead imaging with fibroblast cells at the hydrogel interface. Incorporation of gallic acid imparted antioxidant properties and, along with the degradable network, supported high cytocompatibility (>90% cell viability in Alamar Blue assays). The material gradually resorbed over around 3 weeks in vitro, limiting long‐term accumulation while maintaining performance during the critical healing period. By integrating cationic charge‐mediated coagulation, bioinspired tissue adhesion, antimicrobial drug delivery, and tunable mechanical and degradation profiles, the Cl‐Plu‐Cl/PDMA/PEI(GA) hydrogel addresses key limitations of conventional hemostatic agents, such as poor injectability and weak wet adhesion. It thus presents a versatile platform with strong translational potential as an injectable hemostat, tissue adhesive, antimicrobial drug delivery system, and, potentially, a 3D‐processable scaffold for advanced trauma care and surgical applications.

## Author Contributions

**Arvind K. Singh Chandel**: Writing – review and editing, Writing – original draft, Visualization, Validation, Project administration, Methodology, Investigation, Data curation, and Data analysis, Conceptualization. **Runali Patil**: Investigation. **Abrar Ali Khan**: Investigation. **Deeksha Pandit,** Investigation. **Kaushik Chatterjee** Supervision, Methodology, Investigation **Maurice N Collins**: Writing – review and editing, Visualization, Validation, Supervision, Project administration, Methodology, Investigation, Formal analysis, Data curation, Conceptualization.

## Ethics Approval and Consent to Participate

All animal procedures were approved by the Experimental Animal Welfare and Ethics Committee of the Central Animal Facility, Department of Materials Engineering, Indian Institute of Science, Bangalore, India. Written informed consent was obtained from all human participants before the study in accordance with ethical guidelines.

## Conflicts of Interest

The authors declare no conflicts of interest.

## Supporting information




**Supporting File 1**: adhm70829‐sup‐0001‐MovieS1.mp4.

**Supporting File 2**: adhm70829‐sup‐0002‐MovieS2.mp4.

**Supporting File 3**: adhm70829‐sup‐0003‐MovieS3.mp4.

**Supporting File 4**: adhm70829‐sup‐0004‐MovieS4.mp4.

**Supporting File 5**: adhm70829‐sup‐0005‐MovieS5.mp4.

**Supporting File 6**: adhm70829‐sup‐0006‐MovieS6.mp4.

**Supporting File 7**: adhm70829‐sup‐0007‐SuppMat.docx.

## Data Availability

The data that support the findings of this study are available from the corresponding author upon reasonable request.
