# Supplementary material for: Injectable Stimuli‐Responsive Amphiphilic Hydrogel for Rapid Hemostasis, Robust Tissue Adhesion, and Controlled Drug Delivery in Trauma and Surgical Care
Source: Adv Healthc Mater. 2026 Jan 30;15(16):e05307. doi: 10.1002/adhm.202505307 (PMC13107930; doi:10.1002/adhm.202505307)
Supplement: Supplementary file 7 — Supporting File 7: adhm70829‐sup‐0007‐SuppMat.docx. [file ADHM-15-0-s002.docx]

**Injectable Stimuli‑Responsive Amphiphilic Hydrogel for Rapid Hemostasis, Robust Tissue Adhesion, and Controlled Drug Delivery in Trauma and Surgical Care**

Arvind K. Singh Chandel^*1^, Runali Patil^2^, Abrar Ali khan^1^, Deeksha Pandit^3^, Kaushik Chatterjee^2^ and Maurice N Collins*^1^

^1^School of Engineering, Bernal Institute, University of Limerick, Limerick V94 T9PX, Ireland

^2^Department of Materials Engineering, Indian Institute of Science, Bangalore, India

^3^Central Animal Facility, Department of Materials Engineering, Indian Institute of Science, Bangalore, India

**Corresponding author**

**Maurice N. Collins**

School of Engineering, Bernal Institute,

University of Limerick, Limerick V94 T9PX, Ireland

Email: [maurice.collins@ul.ie](mailto:maurice.collins@ul.ie)

**Determination of grafting ratio of PEI–GA**

^1^H NMR spectra of gallic acid, PEI, and PEI–GA were recorded in D₂O at 400 MHz (Fig. S1(a)). For PEI–GA, the aromatic protons of gallic acid appeared at 6.8–7.2 ppm (signals b–d), while the PEI backbone protons were observed as a broad envelope at 2.5–3.5 ppm (signal a). The degree of substitution (DS, mol GA per mol amine unit) was calculated using:​

$$\text{DS}\text{(\%)}=\frac{\left( \frac{I_{\text{Ar}}}{n_{\text{Ar}}} \right)}{\left( \frac{I_{\text{PEI}}}{n_{\text{PEI}}} \right)} X 100$$

where $I_{\text{Ar}}$ is the integrated area of the gallic‑acid aromatic peaks, $n_{\text{Ar}}$ is the number of aromatic protons per GA, $I_{\text{PEI}}$ is the integral of the PEI backbone protons region, and $n_{\text{PEI}}=4$ is the number of methylene protons in one –CH₂–CH₂–NH– amine unit. Using the integrals, DS was determined to be ~0.21, corresponding to approximately 21% of primary amines substituted with gallic acid.​

To validate this value, UV–Vis spectra of PEI–GA solutions were recorded in water (Fig. S1(b)). Gallic acid exhibits a characteristic absorption at ~290-300 nm. A calibration curve was constructed from known GA standards (0–100 μg mL⁻¹), yielding the linear relation. $A=a+bC$ with $R^{2}>0.99$ (Figure S1c). The GA content in a known PEI–GA solution was back‑calculated from its absorbance at λ_max_ and converted to DS using the PEI repeat‑unit molar concentration. This UV-based DS (19.4%) agreed well with the NMR result, confirming the grafting ratio.

**Determination of end‑group functionalization of Cl‑Plu‑Cl**

The degree of substitution (DS) of the terminal hydroxyl groups of Pluronic F‑127 with 4‑chloromethylbenzoyl units was quantified by ^1^H NMR spectroscopy (CDCl_3_, 400 MHz). In the spectrum of Cl‑Plu‑Cl, the aromatic protons of the 4‑chloromethylbenzoyl groups appeared at 7.4–8.0 ppm (signals c and d), while the PPO methyl protons of the Pluronic backbone were observed at 1.05–1.15 ppm (signal a), and the PPO/PEO methylene protons at 3.2–3.8 ppm (signal b). The integral of the aromatic region was $I_{\text{aryl}}\approx8.0$, and the PPO methyl region gave $I_{\text{CH3}}\approx194.5$, corresponding to ~65 PPO units and confirming the expected F‑127 composition.​ Each 4-chloromethylbenzoyl group contributes four aromatic protons, and each Pluronic F-127 chain has two terminal hydroxyl groups available for esterification. The DS was therefore calculated using:

$$\text{DS (\%)}=\frac{\left( I_{\text{aryl}}/4 \right)}{2}\times100$$

where $I_{\text{aryl}}$ is the total integral of the aromatic protons from the benzoyl groups, 4 is the number of aromatic protons per substituent, and 2 is the number of terminal –OH groups per F‑127 chain. Substituting the experimental value $I_{\text{aryl}}=8.0$ gives:

$$I_{\text{aryl}}/4=8.0/4=2.0$$

$$\text{DS (\%)}=\frac{2.0}{2}\times100=100\%$$

Thus, within experimental error, both PEO termini are fully esterified, and the degree of chloromethylbenzoyl functionalization is ~99–100% per Pluronic F‑127 chain. This confirms that Cl‑Plu‑Cl acts as an essentially quantitatively difunctional crosslinker in the hydrogel network.

**FIGURE S1.** (a) ¹H NMR spectrum of PEI–GA in D₂O, (b) UV–Vis spectrum of PEI–GA, (c) calibration curve of gallic acid used for DS determination, and (d) ¹H NMR spectrum of Cl‑Plu‑Cl in CDCl₃.

**FIGURE 2.** Schematic illustration of the cell culture experimental setup for the hydrogels


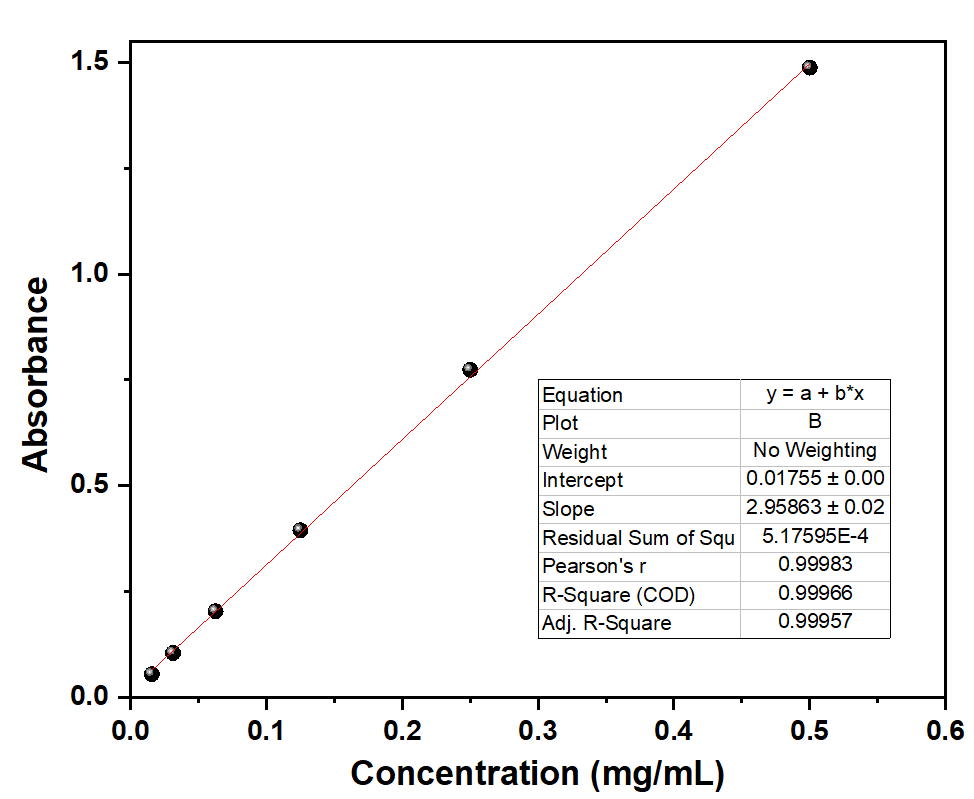


**FIGURE** **S3,** Calibration curve of Amoxicillin in water by using a UV-vis spectrometer


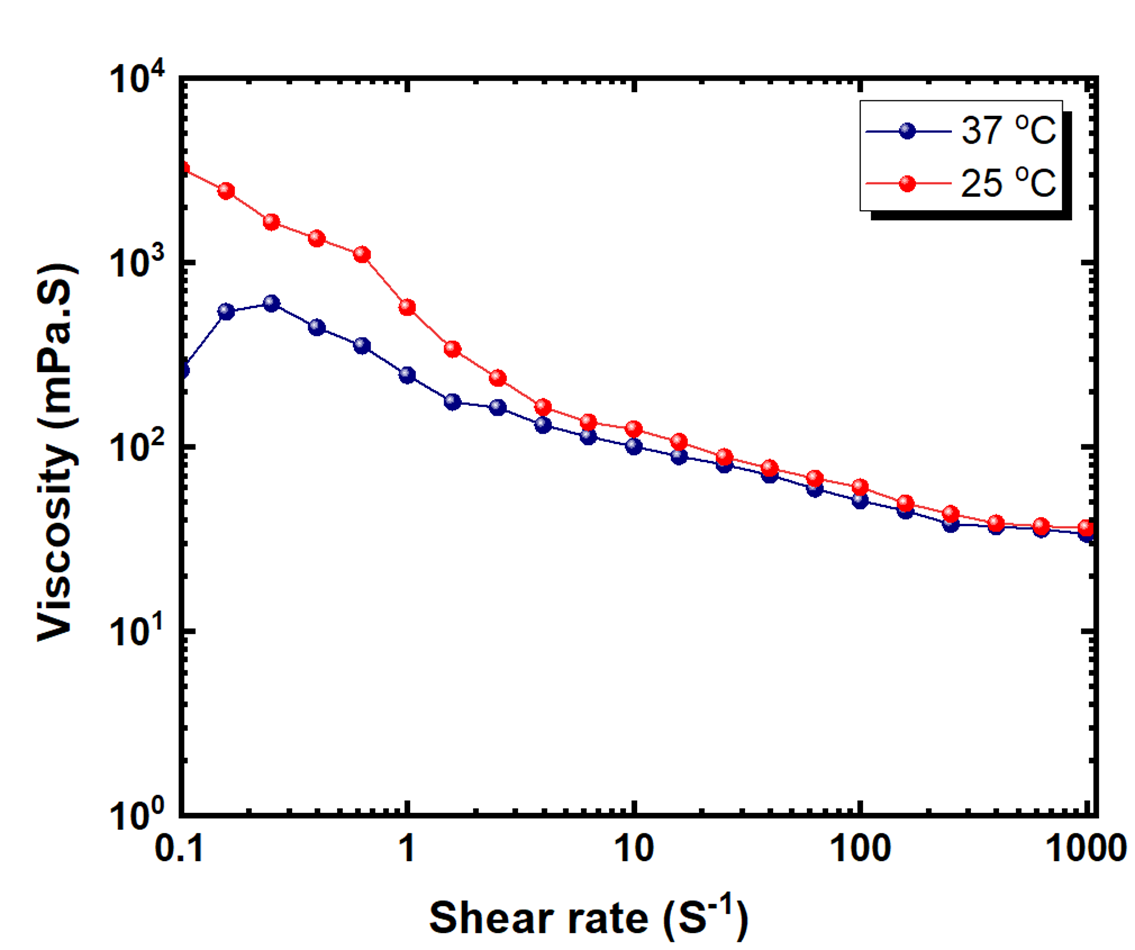


**FIGURE S4** Flow curve of the prepolymer solution unmodified Pluronic F-127 Plu/PDMA/PEI(GA).

**FIGURE S5.** Time-dependent gelation kinetics of different hydrogels, illustrating the differences in gelation time at 25°C (a) Gel-2, (b) Gel-3, and (c) Gel-4

**FIGURE S6.** Frequency‑sweep and strain‑cycling rheological behavior of the hydrogels. Frequency‑sweep curves of storage and loss moduli for (a) Gel‑2, (b) Gel‑3, and (c) Gel‑4 measured at γ = 5%. Alternating strain‑sweep tests on freshly prepared (d) Gel‑2, (e) Gel‑3, and (f) Gel‑4 subjected to repeated low (0.1%) and high (200%) strains for 2 min per step at ω = 6.3 rad s⁻¹, illustrating reversible disruption and recovery of the network structure.


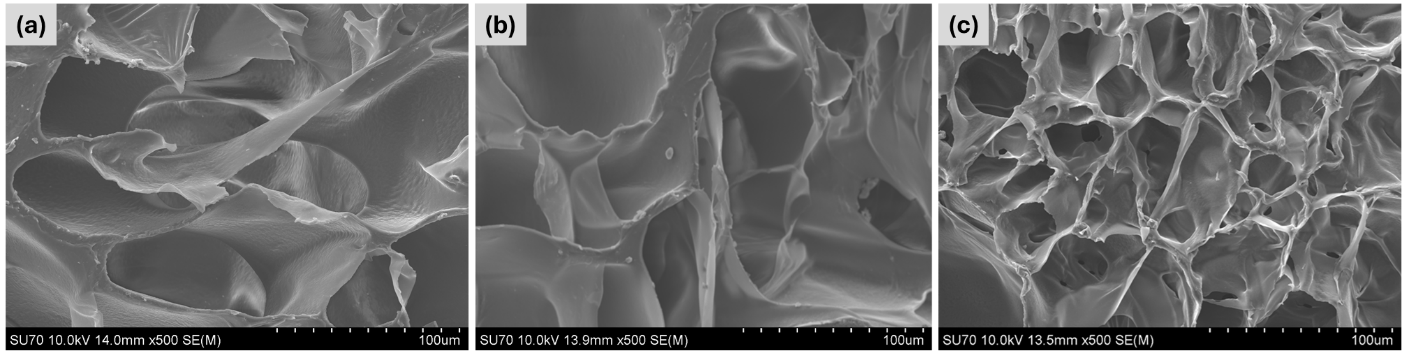


**
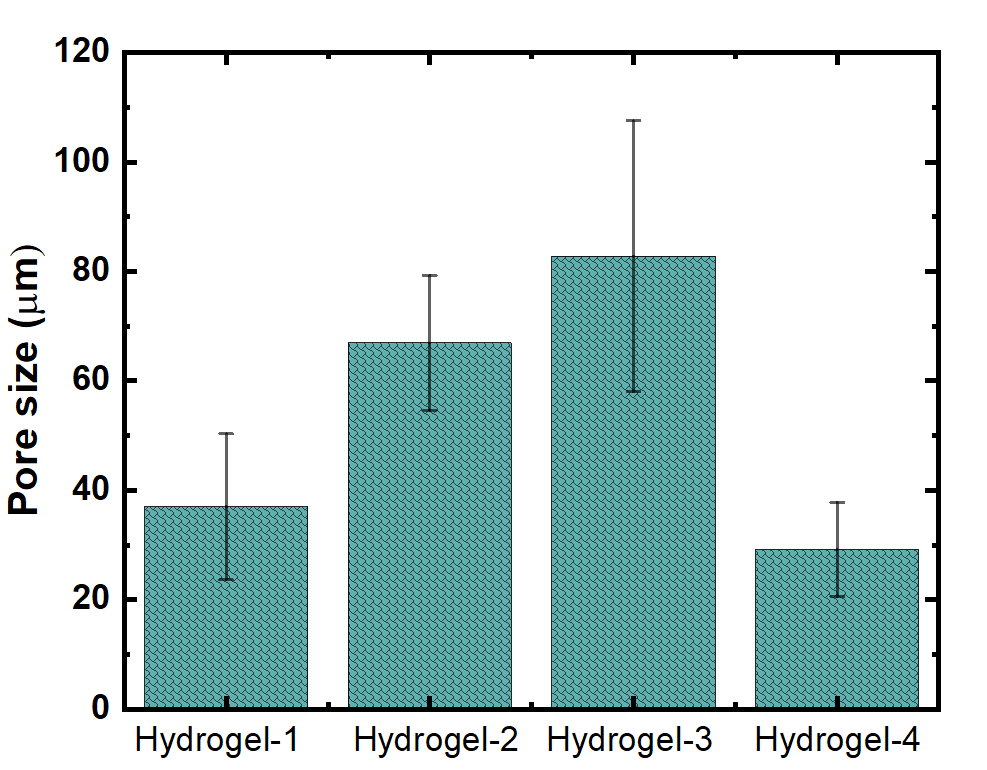
**

**(d)**

**FIGURE S7** SEM images of Hydrogels (a) Hydrogel-2, (b) Hydrogel-3, (c) Hydrogel-4, and comparative pore size graph of all four hydrogels, estimated by SEM images of equilibrated swollen and freeze-dried h

**FIGURE S8.** Cytocompatibility and hemocompatibility of hydrogel degradation products. (a) FT-IR spectra of the intact Cl-Plu-Cl/PDMA/PEI(GA) hydrogel and the recovered degradation mass after in vitro degradation. The intact gel shows a strong ester/amide carbonyl band at approximately 1700–1640 cm⁻¹ (blue region), N–H bending/C–N stretching at approximately 1540 cm⁻¹ (purple), and pronounced C–O–C and C–O bands at approximately 1200–1100 cm⁻¹ (green), consistent with PEG, PDMA, and GA-mediated crosslinks. In the degraded mass, the ester/amide carbonyl peak shifts and decreases in intensity (new band around 1735 cm⁻¹ with reduced signal near 1700–1640 cm⁻¹), while the C–O/C–N bands at approximately 1200–1100 cm⁻¹ become more prominent, indicating hydrolytic cleavage of crosslinks into PEG- and GA-rich oligomeric fragments without formation of new, potentially toxic functionalities. (b) Cell viability of (L-929) fibroblasts cultured with degradation product extracts at 100–1000 µg mL⁻¹ remains above 95 %, comparable to the untreated control, confirming excellent cytocompatibility. (c) Hemolysis assay of human red blood cells incubated with degradation products shows less than 5 % hemolysis at 100–500 µg mL⁻¹ and approximately 8 % at 1000 µg mL⁻¹, all below the 10 % threshold for hemocompatibility, as confirmed by visual inspection of the supernatants.Data in (b,c) are presented as mean ± standard deviation (n = 4).

**FIGURE S9.** (A) Calibration curve for gallic acid-mediated DPPH free radical scavenging, depicting the linear decrease in remaining DPPH absorbance at 517 nm as a function of gallic acid concentration (0–100 μg mL⁻¹; equation: y = 0.04786x + 0.07486, R² = 0.9968). (B) UV-Vis absorption spectra (450–700 nm) of DPPH solutions (25 μM) following 30 min incubation with prepolymers (PDMA, Cl-PGlu-Cl, PEI-GA) and PEI-GA-containing injectable hydrogels at varying crosslinker concentrations (Gel-1 to Gel-4; 10–50 mg mL⁻¹), compared to the gallic acid positive control. Inset photographs of reaction vials (a–i) show visual decolorization from purple (unscavenged DPPH) to yellow (scavenged DPPH-H), confirming dose-dependent antioxidant efficacy.


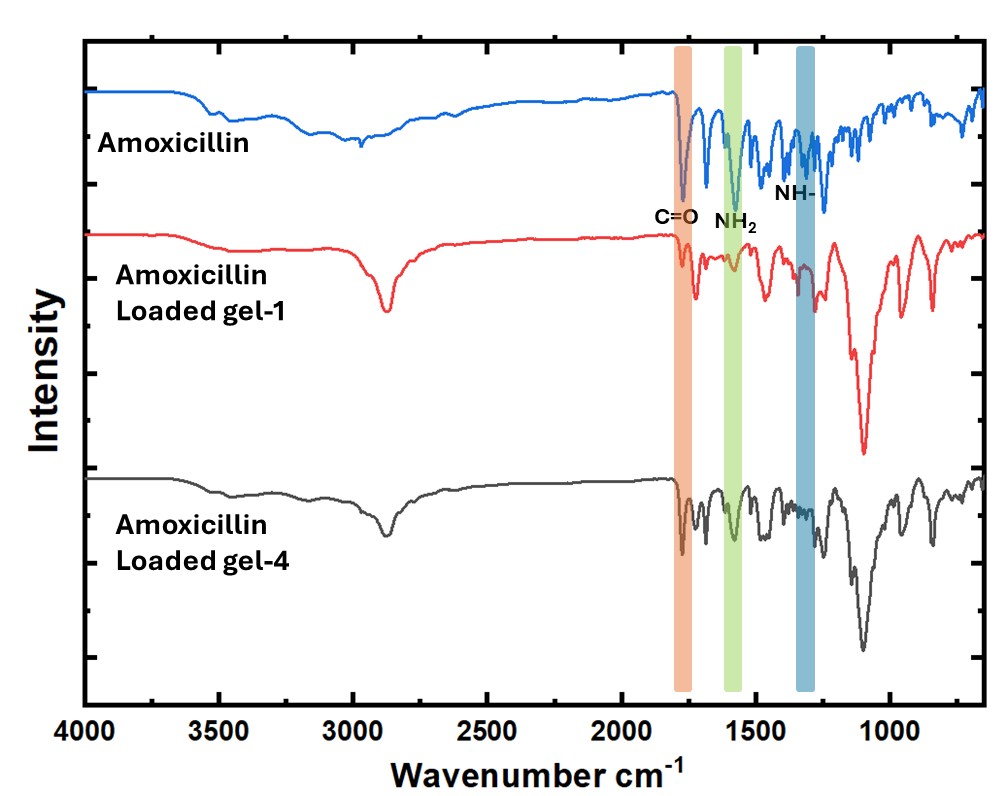


**(a)**

**(b)**

**FIGURE S10.** (a) FT-IR spectra of a representative amoxicillin-loaded Hydrogel-1, Hydrogel-2, and Amoxicillin neat. (B) UV-Visible spectra showing absorbance maxima of neat Amoxicillin dissolved in water and the released Amoxicillin from the hydrogel.

**In vitro antibacterial activity of amoxicillin‑loaded hydrogels**

The antibacterial efficacy of the amoxicillin-loaded Cl-PLu-Cl/PDMA/PEI(GA) hydrogels was tested against *Staphylococcus aureus* and methicillin-resistant *S. aureus* (MRSA) using both a bacterial-killing assay in suspension and an agar diffusion (zone of inhibition) test. For the suspension test, cylindrical hydrogel samples (200 mg) were placed into 2 mL microcentrifuge tubes or 24-well plates containing 1.8 mL of sterile phosphate-buffered saline (PBS). A 200 µL sample of bacterial suspension (∼10⁵–10⁷ CFU mL⁻¹ of S. aureus or MRSA in PBS) was added to each tube, and the mixtures were incubated at 37 °C for 1 hour with gentle shaking. When needed, 100 µL of PBS was added to ensure the gels were fully wet. After incubation, 50 µL of the bacterial suspension was taken, serially diluted 10-fold in PBS, and spread onto nutrient agar plates. These plates were incubated at 37 °C for 24 hours, and the colonies were counted to determine the number of viable bacteria, expressed as log CFU mL⁻¹. Controls included bacterial suspensions incubated in wells without hydrogels and blank hydrogels without amoxicillin.

For the agar diffusion test, nutrient agar plates (90 mm) were prepared by dissolving 2.5 g of nutrient agar in 100 mL of deionized water, autoclaving at 121 °C for 15–18 minutes, and then pouring 12–15 mL of molten agar into each Petri dish. After cooling to room temperature, 0.2 mL of a 10⁸ CFU mL⁻¹ suspension of S. aureus or MRSA was evenly spread over the agar surface. Amoxicillin-loaded hydrogel discs (~100 mg) were placed at the center of each plate, which were then incubated at 37 °C for 12 hours. The diameter of the clear zone of bacterial growth inhibition surrounding each hydrogel was measured with a digital caliper and used as a measure of antibacterial activity.

**FIGURE S11**. In vitro antibacterial activity of amoxicillin‑loaded hydrogels. (a) Representative agar plates showing bacterial growth of *S. aureus* and MRSA in the absence (top) and presence (bottom) of amoxicillin‑loaded hydrogels, illustrating clear zones of inhibition only around drug‑loaded samples. (b) Quantitative bacterial viability for *S. aureus* expressed as CFU mL⁻¹ after 1 h incubation with control (bacteria only), blank hydrogel, and amoxicillin‑loaded hydrogel. (c) Quantitative bacterial viability for MRSA under the same conditions, demonstrating a pronounced reduction in CFU mL⁻¹ for the amoxicillin‑loaded hydrogel compared with control and blank hydrogel groups.

**FIGURE S12.** pH-dependent mechanical and adhesive performance of Cl‑Plu‑Cl/PDMA/PEI(GA) hydrogels. (a) Compressive Young’s modulus of the optimized Plu‑Cl/PDMA/PEI(GA) Gel‑4 at pH 7.4 and pH 5.0, calculated from the linear region (15–25 % strain) of the uniaxial compression stress–strain curves; gels pre-equilibrated in PBS (pH 7.4) or acetate buffer (pH 5.0) for 24 h before testing. (b) Representative compression force–displacement curves for Gel‑4 at pH 7.4 and pH 5.0, showing higher peak force and stiffness under mildly acidic conditions, consistent with protonation-enhanced ionic interactions within the network. (c) Lap‑shear adhesion strength of Gel‑4 on sausage-casing tissue at pH 7.4 and pH 5.0, measured using a modified ASTM F2255-05 protocol; adhesion remains high and only modestly reduced at pH 5.0. (d) Representative adhesion strength–displacement curves for Gel‑4 at pH 7.4 and pH 5.0, illustrating robust interfacial toughness and similar failure profiles under both conditions. Data in (a,c) are presented as mean ± SD (n=4).

| **TABLE S1**. Comparison of commercially available, clinically used hemostatic adhesives and our hydrogels | | | | | | |
| --- | --- | --- | --- | --- | --- | --- |
| *Sr No.* | **Commercial Gel** | **Advantages** | **Burst pressure (mmHg)** | **Adhesion Strength (kPa)** | **Disadvantages** | **Ref.** |
| *1.* | FLOSEAL (gelatin + thrombin) | Fast hemostasis, easy to apply, works in wet fields | Study Not Available | Study Not Available | Expensive, risk of viral transmission (plasma-derived), no drug delivery function | [1] |
| *2.* | SURGIFLO (gelatin + thrombin) | Flowable, effective in irregular wounds, easy reconstitution | 15-25 | 0.2-0.25 | Limited mechanical strength, degrades relatively fast | [2, 3] |
| *3.* | Tisseel / Tissucol (fibrin sealant) | Excellent tissue adhesion, widely used, biocompatible | 15 on lungs and ≈10–20 in collagen | 5–15 | Slow clot formation compared to gelatin-thrombin, risk of infection, and costly | [4] |
| *4.* | Coseal (Two synthetic PEGs (multi-arm PEG‑NHS systems) + buffer, forming PEG hydrogel. | Strong adhesion, forms a barrier, and is biocompatible | 207 ± 29 | 10–20 | Expensive, no intrinsic hemostatic activity, no drug delivery | [5] |
| *5* | BioGlue (albumin–glutaraldehyde) | Very high mechanical strength and burst resistance; widely used in vascular surgery | 596 ± 72 | 400-485 | Potential for inflammatory response and tissue necrosis; not resorbable; rigid | [1, 6] |
| *6* | Tisseel / Evicel (fibrin sealants)Human fibrinogen + thrombin (plasma-derived or recombinant) | Biocompatible, resorbable, long clinical track record | 10 ± 5 | 5–15 | Relatively low mechanical strength; slower clotting than gelatin–thrombin; blood-derived components | [7] |
| *7* | Progel (PEG–human serum albumin)PEG–NHS crosslinked with human serum albumin | Approved for lung sealant; good elasticity and sealing in pulmonary surgery | 76.3 ± 15.8 | 78.7 ± 14 | Lower burst pressure than some engineered hydrogels; limited intrinsic hemostasis | [8] |
| *8* | Vistaseal (fibrin sealant)Human fibrinogen + thrombin (dual‑syringe, spray) | Good handling; similar indications to Tisseel | 12.6- 300 | 8-30 | Limited mechanical strength, similar to other fibrin glues | [9] |
| *9* | Beriplast® Fibrinogen + Factor XIII (+ aprotinin)  Thrombin + calcium chloride | Clinically approved and widely used, with excellent hemostatic efficacy and high biocompatibility. Biodegradable and resorbable, easy to apply, with rapid clot formation. | 25-60 | 2-3 | Low mechanical strength/burst pressure, not suitable for high-pressure arterial sealing, short residence time (enzymatic degradation), and limited adhesion on wet or dynamic tissues. | [10] |
| *10* | Cl‑Plu‑Cl/PDMA/PEI(GA) (this work) | Strong wet‑tissue adhesion and burst resistance; injectable, pH-responsive, ROS-scavenging, and antibiotic-releasing hydrogel, with tunable degradation. | ≈25–30 kPa on wet porcine skin) | ≈50–220 on porcine skin | Currently investigational; long-term clinical safety and performance not yet established |  |

**TABLE S2** Drug Release Kinetics and Mathematical Models Fitted Data of In Vitro Amoxicillin Release from Various Hydrogels at pH 7.4 and 5

| Hydrogels | Release  medium | Zero Order | | First Order | | Higuchi model | | Kors Meyar  Peppas | | Hixon  Crowell | |
| --- | --- | --- | --- | --- | --- | --- | --- | --- | --- | --- | --- |
|  |  | R^2^ | Slope | R^2^ | Slope | R^2^ | Slope | R^2^ | Slope | R^2^ | Slope |
| Gel-1 | pH 7.4 | 0.9954 | 0.3369 | 0.9675 | -0.0024 | 0.9367 | 4.410 | 0.885 | 0.3266 | 0.9814 | -0.0073 |
| Gel-1 | pH 5 | 0.9962 | 0.4995 | 0.8694 | -0.006 | 0.9618 | 6.622 | 0.935 | 0.3994 | 0.9507 | -0.0144 |
| Gel-2 | pH 7.4 | 0.9975 | 0.4332 | 0.9447 | -0.0039 | 0.9496 | 5.703 | 0.897 | 0.3702 | 0.9758 | -0.0106 |
| Gel-2 | pH 5 | 0.9737 | 0.5164 | 0.8904 | -0.0091 | 0.9887 | 7.020 | 0.979 | 0.3633 | 0.9683 | -0.0183 |
| Gel-3 | pH 7.4 | 0.9989 | 0.5174 | 0.7811 | -0.0083 | 0.9466 | 6.796 | 0.893 | 0.3601 | 0.9196 | -0.0171 |
| Gel-3 | pH 5 | 0.9134 | 0.5031 | 0.9293 | -0.0131 | 0.9866 | 7.062 | 0.994 | 0.3241 | 0.9817 | -0.0219 |
| Gel-4 | pH 7.4 | 0.9977 | 0.3620 | 0.9793 | -0.0027 | 0.9429 | 4.748 | 0.877 | 0.3372 | 0.9897 | -0.0080 |
| Gel-4 | pH 5 | 0.9865 | 0.5123 | 0.9023 | -0.0066 | 0.9845 | 6.906 | 0.985 | 0.4079 | 0.9703 | -0.0155 |

**References**

1. Murdock, M.H., et al., *Cytocompatibility and mechanical properties of surgical sealants for cardiovascular applications.* The Journal of thoracic and cardiovascular surgery, 2018 Sep 1. **157**(1).

2. Feng, G., et al., *Bench-to-bedside translation of Self-Healing colloidal hydrogels as 2nd generation design of Flowable Hemostatic Matrix: From Preclinical evaluation to Human Clinical Trials.* medRxiv, 2025-11-28.

3. Hong, Y., et al., *A strongly adhesive hemostatic hydrogel for the repair of arterial and heart bleeds.* Nature Communications, 2019 May 14. **10**(1).

4. TB, P., et al., *Comparative study of lung sealants in a porcine ex vivo model - PubMed.* The Annals of thoracic surgery, 2012 Jul. **94**(1).

5. Kühlbrey, C.M., et al., *<em>Journal of Hepato-Biliary-Pancreatic Sciences</em> | JSHBPS Pancreas Journal | Wiley Online Library.* Journal of Hepato-Biliary-Pancreatic Sciences, 2019/03/01. **26**(3).

6. Eshkol-Yogev, I., et al., *Dual composite bioadhesives for wound closure applications: An in vitro and in vivo study.* Polymers for Advanced Technologies, 2022/11/01. **33**(11).

7. Chen, L.X., et al., *Investigation of surgical adhesives for vocal fold wound closure.* The Laryngoscope, 2019/09/01. **129**(9).

8. Fuller, C., *Reduction of intraoperative air leaks with Progel in pulmonary resection: a comprehensive review.* Journal of Cardiothoracic Surgery, 2013 Apr 16. **8**.

9. L, L., et al., *Tensile strength of biological fibrin sealants: a comparative study - PubMed.* The Journal of surgical research, 2012 Aug. **176**(2).

10. Tsai, C.-C., et al., *Injectable, Shear-Thinning, Self-Healing, and Self-Cross-Linkable Benzaldehyde-Conjugated Chitosan Hydrogels as a Tissue Adhesive.* Biomacromolecules, January 30, 2024. **25**(2).
